# A counterfactual approach to bias and effect modification in terms of response types

**DOI:** 10.1186/1471-2288-13-101

**Published:** 2013-07-31

**Authors:** Etsuji Suzuki, Toshiharu Mitsuhashi, Toshihide Tsuda, Eiji Yamamoto

**Affiliations:** 1 Department of Epidemiology, Graduate School of Medicine, Dentistry and Pharmaceutical Sciences, Okayama University, 2-5-1 Shikata-cho, Kita-ku, Okayama 700-8558, Japan; 2 Department of Human Ecology, Graduate School of Environmental and Life Science, Okayama University, 3-1-1 Tsushima-naka, Kita-ku, Okayama 700-8530, Japan; 3 Department of Information Science, Faculty of Informatics, Okayama University of Science, 1–1 Ridai-cho, Kita-ku, Okayama 700-0005, Japan

**Keywords:** Bias, Causal inference, Counterfactual, Directed acyclic graphs, Effect modification, Exchangeability, Randomization, Response types

## Abstract

**Background:**

The counterfactual approach provides a clear and coherent framework to think about a variety of important concepts related to causation. Meanwhile, directed acyclic graphs have been used as causal diagrams in epidemiologic research to visually summarize hypothetical relations among variables of interest, providing a clear understanding of underlying causal structures of bias and effect modification. In this study, the authors aim to further clarify the concepts of bias (confounding bias and selection bias) and effect modification in the counterfactual framework.

**Methods:**

The authors show how theoretical data frequencies can be described by using unobservable response types both in observational studies and in randomized controlled trials. By using the descriptions of data frequencies, the authors show epidemiologic measures in terms of response types, demonstrating significant distinctions between association measures and effect measures. These descriptions also demonstrate sufficient conditions to estimate effect measures in observational studies. To illustrate the ideas, the authors show how directed acyclic graphs can be extended by integrating response types and observed variables.

**Results:**

This study shows a hitherto unrecognized sufficient condition to estimate effect measures in observational studies by adjusting for confounding bias. The present findings would provide a further understanding of the assumption of conditional exchangeability, clarifying the link between the assumptions for making causal inferences in observational studies and the counterfactual approach. The extension of directed acyclic graphs using response types maintains the integrity of the original directed acyclic graphs, which allows one to understand the underlying causal structure discussed in this study.

**Conclusions:**

The present findings highlight that analytic adjustment for confounders in observational studies has consequences quite different from those of physical control in randomized controlled trials. In particular, the present findings would be of great use when demonstrating the inherent distinctions between observational studies and randomized controlled trials.

## Background

The counterfactual approach provides a clear and coherent framework to think about a variety of important concepts related to causation [1,2]. In particular, the counterfactual approach to confounding has been widely accessible to epidemiologists since the publication of a classic methods paper by Greenland and Robins [3], and the concept of bias is now explained in the counterfactual framework [4-12]. (Note that an update of the classic methods paper was recently published [13]). Meanwhile, directed acyclic graphs (DAGs) have long been used as causal diagrams in epidemiologic research to visually summarize hypothetical relations among variables of interest [14,15]. DAGs have been used extensively to determine the variables for which it is necessary to control for confounding bias to estimate causal effects [14-20]. Besides, Hernán et al. [21] showed that various types of selection bias share a common underlying causal structure, and referred to conditioning on common effects as selection bias. Furthermore, VanderWeele and Robins [22] provided a structural classification of effect modification by using DAGs. Indeed, the different approaches provide complementary perspectives, and can be employed together to provide a clearer understanding of causality [23].

In this study, we aim to further clarify the concepts of bias (confounding bias and selection bias) and effect modification in the counterfactual framework. To achieve this, we show how theoretical data frequencies can be described by using unobservable response types both in observational studies and in randomized controlled trials. These descriptions also demonstrate sufficient conditions to estimate effect measures in observational studies, which would provide a further understanding of the assumption of conditional exchangeability. To illustrate the ideas, DAGs are employed, and we show how one can extend the original DAGs by integrating response types and observed variables. We deal only with structural (systematic) relations among the underlying variables of interest, so that an issue of random variation does not arise. Throughout this article, we assume that the consistency condition is met [24-28].

## Methods

### Definitions and notation

#### A causal diagram and causal effects

**Figure 1:**
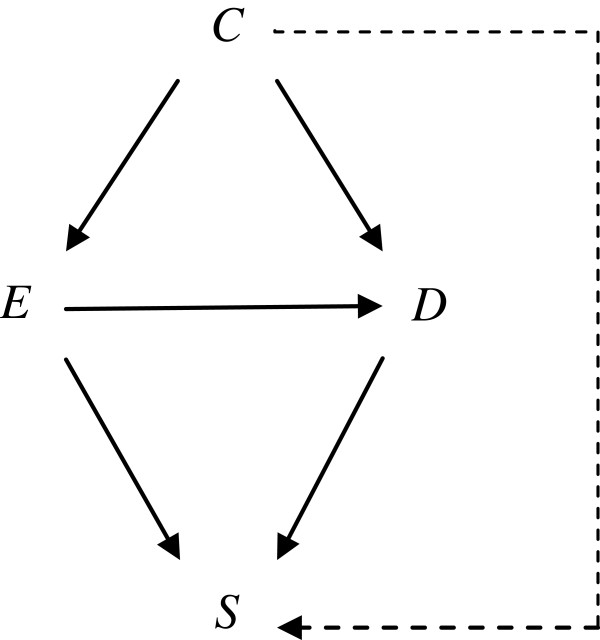
**A causal diagram depicting a hypothetical example.***E*, *D*, *C*, and *S* denote exposure, disease, confounder, and selection variable, respectively. *C* may act as a direct effect modifier simultaneously. In this paper, *S* is assumed not to be influenced directly by *C* (i.e., the dashed arrow is absent). The present discussion, however, can be readily extended to the situations in which one assumes that the dashed arrow is present.

We use a total of 4 binary variables as shown in Figure 1. We let *D* denote a binary outcome of interest (1: outcome occurred, 0: outcome did not occur) and let *E* denote a binary cause of interest (1: exposed, 0: unexposed) that is potentially manipulable. We let *C* denote a binary common cause of *E* and *D* (1: present, 0: absent), which is also potentially manipulable. Typically, *C* is called a confounder of the effect of *E* on *D*. (Note that we assume that *C* precedes *E* temporally in this study, which is in general not necessary for *C* to be a confounder. Recently, VanderWeele and Shpitser [29,30] further discussed the definition of a confounder.) As explained later, *C* can also act as a direct effect modifier for the causal effect of *E* on *D* because *C* is a direct cause of *D*[22]. Finally, we let *S* denote selection variable (1: selected, 0: not selected), which is a common effect of *E* and *D*. Adjustment for *S* yields a spurious association between *E* and *D*, which is called selection bias [21]. Alternatively, one may assume that *S* is also directly influenced by *C*, as shown by using a dashed arrow in Figure 1. Although the arrow is assumed to be absent throughout this paper to avoid technical complications, the following discussion can be readily extended to the situations in which the dashed arrow is present.

Throughout this paper, we discuss the case where the causal effect of primary interest is the effect of *E* on *D* in the total population, including the subpopulations of *C* = 0 and *C* = 1. In the counterfactual framework, this causal effect is given by comparing *P*[*D*_*e*=0_ = 1] and *P*[*D*_*e*=1_ = 1], where *P*[*D*_*e*_ = 1] denotes the proportion of subjects that would have developed the outcome *D* = 1 had all subjects in the total population received exposure value *e*. The variables *D*_*e*=0_ and *D*_*e*=1_ are referred to as potential outcomes. Therefore, when we use risk ratios (RRs) as a measure of interest, a causal RR in the total population is given by

(1) $$\frac{P\left[D_{e=1}=1\right]}{P\left[D_{e=0}=1\right]}.$$

Causal RRs in the total population can be consistently estimated under the assumption of (conditional) exchangeability, or, equivalently, no unmeasured confounding (i.e., *E*∐*D*_*e*_ for ∀*e*). Furthermore, in addition to the effect of *E* on *D* in the total population, we also discuss causal effect of *E* on *D* within the subsets of *C*. In this case, a causal RR within the subsets is given by

(2) $$\frac{P\left[D_{e=1}=1|C=c\right]}{P\left[D_{e=0}=1|C=c\right]}.$$

Causal RRs in the subsets of *C* can be consistently estimated under the assumption of conditional exchangeability, or, equivalently, no unmeasured confounding given data on *C* (i.e., *E*∐*D*_*e*_|*C* for ∀*e*). Note that, when the causal effect of interest is the effect of *E* on *D* either in the total population or in the subsets of *C*, intervening on *E* is of concern, and one does not consider intervening on *C*. Indeed, as outlined by VanderWeele [31], intervening on *C* would only be of concern if the joint effect of *E* and *C* on *D* was of interest. Therefore, under the situation in which *C* is being considered as a potential confounder as well as direct effect modifier (Figure 1), intervening on *C* is not of interest.

When we show how theoretical data frequencies can be described by using unobservable response types in observational studies, however, it is of great use to elucidate the relations between *C* and *E* in the counterfactual framework. By so doing, we demonstrate sufficient conditions to estimate effect measures in observational studies, which would provide a further understanding of the assumption of conditional exchangeability.

#### Response types

First, we elucidate the relations between *C* and *E* by hypothetically conceptualizing potential outcomes of *E* in the counterfactual framework. We let *E*_*c*_(*ω*) denote the potential outcomes of *E* for individual *ω* if, possibly contrary to fact, there had been interventions to set *C* to *c*. (In the following sections, we explicitly show *ω* to discuss response types.) Then, for each individual *ω*, there would be 2 relevant potential outcomes of *E*, i.e., *E*_1_(*ω*) and *E*_0_(*ω*), which correspond to exposure status of that individual when *C* is present and absent, respectively. As a result, individuals can be classified into 4 (i.e., 2^2^) different *E* response types as enumerated in Table 1. We let *E*^T^(*ω*) denote *E* response type of individual *ω*. In some cases, the effect of *C* may be in the same direction for all individuals in the population. We say that *C* has a positive monotonic effect on *E* if *E*_*c*_(*ω*) is non-decreasing in *c* for all individuals, i.e., *E*_1_(*ω*) ≥ *E*_0_(*ω*) for ∀*ω*, which excludes *E* response type 3. Note that this should be clearly distinguished from the assumptions of no preventive action or no preventive sequence [32,33].

**Table 1:** **Enumeration of 4 response types for exposure *E *** **and corresponding potential outcomes**

***E *** **type**	**Potential outcomes of *E***	
***E***^**T**^**( *ω *)**	***E***_***c***_**( *ω *)**	
	***E***_**1**_**( *ω *)**	***E***_**0**_**( *ω *)**
1	1	1
2	1	0
3 ^a^	0	1
4	0	0

^a^ Under the assumption of positive monotonicity (i.e., *E*_1_(*ω*) ≥ *E*_0_(*ω*) for ∀*ω*), this response type is excluded.

In a similar manner, we let *D*_*ce*_(*ω*) denote the potential outcomes of *D* for individual *ω* if, possibly contrary to fact, there had been interventions to set *C* to *c* and to set *E* to *e*. For each individual *ω*, there would thus be 4 possible potential outcomes *D*_11_(*ω*), *D*_01_(*ω*), *D*_10_(*ω*), and *D*_00_(*ω*), resulting in 16 (i.e., 2^4^) different *D* response types as enumerated in Table 2[34]. We let *D*^T^(*ω*) denote *D* response type of individual *ω*. We say that *C* and *E* have positive monotonic effects on *D* if *D*_*ce*_(*ω*) is non-decreasing in *c* and *e* for all individuals, i.e., *D*_*ce*_(*ω*) ≥ *D*_*c* ' *e* '_(*ω*) for ∀*ω* whenever *c* ≥ *c* ' and *e* ≥ *e* '. Under this assumption, the individuals of *D* response types 3, 5, 7, and 9 through 15 are excluded; and individuals of *D* response types 1, 2, 4, 6, 8, and 16 may remain [32].

**Table 2:** **Enumeration of 16 response types for outcome *D *** **and corresponding potential outcomes**

***D *** **type**	**Potential outcomes of *D***			
***D***^**T**^**( *ω *)**	***D***_***ce***_**( *ω *)**			
	***D***_**11**_**( *ω *)**	***D***_**01**_**( *ω *)**	***D***_**10**_**( *ω *)**	***D***_**00**_**( *ω *)**
1	1	1	1	1
2 ^b, c^	1	1	1	0
3 ^a, b, c^	1	1	0	1
4	1	1	0	0
5 ^a, b, c^	1	0	1	1
6	1	0	1	0
7 ^a, b, c^	1	0	0	1
8 ^b^	1	0	0	0
9 ^a, b, c^	0	1	1	1
10 ^a, b, c^	0	1	1	0
11 ^a^	0	1	0	1
12 ^a, b^	0	1	0	0
13 ^a^	0	0	1	1
14 ^a, b^	0	0	1	0
15 ^a, b^	0	0	0	1
16	0	0	0	0

^a^ Under the assumption of positive monotonicity (i.e., *D*_*ce*_(*ω*) ≥ *D*_*c* ' *e* '_(*ω*) for ∀*ω* whenever *c* ≥ *c* ' and *e* ≥ *e* '), these response types are excluded.

^b^ Given no interaction at the individual level on the additive scale between *C* and *E* in the counterfactual framework (i.e., *D*_11_(*ω*) − *D*_01_(*ω*) − *D*_10_(*ω*) + *D*_00_(*ω*) = 0 for ∀*ω*), these response types are excluded.

^c^ Given no interaction at the individual level on the multiplicative scale between *C* and *E* in the counterfactual framework (i.e., *D*_11_(*ω*)*D*_00_(*ω*) = *D*_01_(*ω*)*D*_10_(*ω*) for ∀*ω*), these response types are excluded.

Likewise, we let *S*_*ed*_(*ω*) denote the potential outcomes of *S* for individual *ω* if, possibly contrary to fact, there had been interventions to set *E* to *e* and to set *D* to *d*. For each individual *ω*, there would thus be 4 possible potential outcomes *S*_11_(*ω*), *S*_01_(*ω*), *S*_10_(*ω*), and *S*_00_(*ω*), resulting in 16 (i.e., 2^4^) different *S* response types as enumerated in Table 3. We let *S*^T^(*ω*) denote *S* response type of individual *ω*.

**Table 3:** **Enumeration of 16 response types for selection variable *S *** **and corresponding potential outcomes**

***S *** **type**	**Potential outcomes of *S***			
***S***^**T**^**( *ω *)**	***S***_***ed***_**( *ω *)**			
	***S***_**11**_**( *ω *)**	***S***_**01**_**( *ω *)**	***S***_**10**_**( *ω *)**	***S***_**00**_**( *ω *)**
1	1	1	1	1
2 ^b, c^	1	1	1	0
3 ^a, b, c^	1	1	0	1
4	1	1	0	0
5 ^a, b, c^	1	0	1	1
6	1	0	1	0
7 ^a, b, c^	1	0	0	1
8 ^b^	1	0	0	0
9 ^a, b, c^	0	1	1	1
10 ^a, b, c^	0	1	1	0
11 ^a^	0	1	0	1
12 ^a, b^	0	1	0	0
13 ^a^	0	0	1	1
14 ^a, b^	0	0	1	0
15 ^a, b^	0	0	0	1
16	0	0	0	0

^a^ Under the assumption of positive monotonicity (i.e., *S*_*ed*_(*ω*) ≥ *S*_*e* ' *d* '_(*ω*) for ∀*ω* whenever *e* ≥ *e* ' and *d* ≥ *d* '), these response types are excluded.

^b^ Given no interaction at the individual level on the additive scale between *E* and *D* in the counterfactual framework (i.e., *S*_11_(*ω*) − *S*_01_(*ω*) − *S*_10_(*ω*) + *S*_00_(*ω*) = 0 for ∀*ω*), these response types are excluded.

^c^ Given no interaction at the individual level on the multiplicative scale between *E* and *D* in the counterfactual framework (i.e., *S*_11_(*ω*)*S*_00_(*ω*) = *S*_01_(*ω*)*S*_10_(*ω*) for ∀*ω*), these response types are excluded.

Finally, we integrate information about the potential outcomes discussed above to produce 2 types of compound potential outcomes, which are also called nested counterfactuals [2]. (Note that compound potential outcomes have been extensively used in the issues of mediation and direct/indirect effects [35-38].) First, we combine the potential outcomes of *E* and the potential outcomes of *D* to define $D_{cE_{c'}}\left(\omega \right)$. In other words, the compound potential outcomes of *D* are defined by (i) confounder status (*C*(*ω*) = 1, *C*(*ω*) = 0) and (ii) potential exposure status following an intervention on confounder (*E*_1_(*ω*), *E*_0_(*ω*)). For each individual *ω*, there would thus be 4 possible compound potential outcomes $D_{1E_{1}}\left(\omega \right)$, $D_{1E_{0}}\left(\omega \right)$, $D_{0E_{1}}\left(\omega \right)$, and $D_{0E_{0}}\left(\omega \right)$. Second, we combine the potential outcomes of *E*, the potential outcomes of *D*, and the potential outcomes of *S* to define $S_{E_{c}D_{c'E_{c''}}}\left(\omega \right)$. Note that the compound potential outcomes of *S* are defined by (i) potential exposure status following an intervention on confounder (*E*_1_(*ω*), *E*_0_(*ω*)) and (ii) the compound potential outcomes of *D* ($D_{1E_{1}}\left(\omega \right)$, $D_{1E_{0}}\left(\omega \right)$, $D_{0E_{1}}\left(\omega \right)$, and $D_{0E_{0}}\left(\omega \right)$). Thus, for each individual *ω*, there would be 8 possible compound potential outcomes $S_{E_{1}D_{1E_{1}}}\left(\omega \right)$, $S_{E_{1}D_{1E_{0}}}\left(\omega \right)$, $S_{E_{1}D_{0E_{1}}}\left(\omega \right)$, $S_{E_{1}D_{0E_{0}}}\left(\omega \right)$, $S_{E_{0}D_{1E_{1}}}\left(\omega \right)$, $S_{E_{0}D_{1E_{0}}}\left(\omega \right)$, $S_{E_{0}D_{0E_{1}}}\left(\omega \right)$, and $S_{E_{0}D_{0E_{0}}}\left(\omega \right)$.

Combination of 4 *E* response types, 16 *D* response types, and 16 *S* response types yields 1,024 (i.e., 4 × 16 × 16) *EDS* response types. As noted above, under the assumption of positive monotonic effect of *C* on *E*, the number of possible *E* response types is reduced from 4 to 3. Further, under the assumptions of both positive monotonic effects of *C* and *E* on *D* and no interaction at the individual level on the additive scale between *C* and *E* on *D*, the number of possible *D* response types is reduced from 16 to 4 (see footnote of Table 2). Analogous argument applies to *S* response types (see footnote of Table 3). Consequently, the number of possible *EDS* response types is reduced from 1,024 to 48 (i.e., 3 × 4 × 4). In Table 4, we show a complete enumeration of these 48 *EDS* response types. To enhance readability, Table 4 shows only selection status when *C* = 1 (i.e., $S_{E_{1}D_{1E_{1}}}\left(\omega \right)$) and when *C* = 0 (i.e., $S_{E_{0}D_{0E_{0}}}\left(\omega \right)$) among $S_{E_{c}D_{c'E_{c''}}}\left(\omega \right)$. Note that we made these restrictive assumptions to show the correspondence between *E* response types, *D* response types, and *S* response types in Table 4, which would be of great help to understand the present findings. The following discussion however applies even without these assumptions. Thus, in the following sections, we use a total of 1,024 *EDS* response types, considering general cases in which these assumptions are not met.

**Table 4:** **Enumeration of 48 *EDS *** **response types and corresponding potential outcomes**

***E *** **type**	***D *** **type**	***S *** **type**	**Potential outcomes of *E***		**Potential outcomes of *D***				**Compound potential outcomes of *D***				**Potential outcomes of *S***				**Selection status**	
***E***^**T**^**( *ω *)**	***D***^**T**^**( *ω *)**	***S***^**T**^**( *ω *)**	***E***_***c***_**( *ω *)**		***D***_***ce***_**( *ω *)**				$D_{cE_{c'}}\left(\omega \right)$				***S***_***ed***_**( *ω *)**				$S_{E_{c}D_{cE_{c}}}\left(\omega \right)$	
			***E***_**1**_	***E***_**0**_	***D***_**11**_	***D***_**01**_	***D***_**10**_	***D***_**00**_	$D_{1E_{1}}$	$D_{1E_{0}}$	$D_{0E_{1}}$	$D_{0E_{0}}$	***S***_**11**_	***S***_**01**_	***S***_**10**_	***S***_**00**_	$S_{E_{1}D_{{}_{1}E_{{}_{1}}}}$	$S_{E_{0}D_{{}_{0}E_{{}_{0}}}}$
1	1	1	1	1	1	1	(1)^a^	(1)	1	(1)	(1)	1	1	(1)	(1)	(1)	1	1
1	1	4	1	1	1	1	(1)	(1)	1	(1)	(1)	1	1	(1)	(0)	(0)	1	1
1	1	6	1	1	1	1	(1)	(1)	1	(1)	(1)	1	1	(0)	(1)	(0)	1	1
1	1	16	1	1	1	1	(1)	(1)	1	(1)	(1)	1	0	(0)	(0)	(0)	0	0
1	4	1	1	1	1	1	(0)	(0)	1	(1)	(1)	1	1	(1)	(1)	(1)	1	1
1	4	4	1	1	1	1	(0)	(0)	1	(1)	(1)	1	1	(1)	(0)	(0)	1	1
1	4	6	1	1	1	1	(0)	(0)	1	(1)	(1)	1	1	(0)	(1)	(0)	1	1
1	4	16	1	1	1	1	(0)	(0)	1	(1)	(1)	1	0	(0)	(0)	(0)	0	0
1	6	1	1	1	1	0	(1)	(0)	1	(1)	(0)	0	1	(1)	1	(1)	1	1
1	6	4	1	1	1	0	(1)	(0)	1	(1)	(0)	0	1	(1)	0	(0)	1	0
1	6	6	1	1	1	0	(1)	(0)	1	(1)	(0)	0	1	(0)	1	(0)	1	1
1	6	16	1	1	1	0	(1)	(0)	1	(1)	(0)	0	0	(0)	0	(0)	0	0
1	16	1	1	1	0	0	(0)	(0)	0	(0)	(0)	0	1	(1)	1	(1)	1	1
1	16	4	1	1	0	0	(0)	(0)	0	(0)	(0)	0	1	(1)	0	(0)	0	0
1	16	6	1	1	0	0	(0)	(0)	0	(0)	(0)	0	1	(0)	1	(0)	1	1
1	16	16	1	1	0	0	(0)	(0)	0	(0)	(0)	0	0	(0)	0	(0)	0	0
2	1	1	1	0	1	(1)	(1)	1	1	(1)	(1)	1	1	1	(1)	(1)	1	1
2	1	4	1	0	1	(1)	(1)	1	1	(1)	(1)	1	1	1	(0)	(0)	1	1
2	1	6	1	0	1	(1)	(1)	1	1	(1)	(1)	1	1	0	(1)	(0)	1	0
2	1	16	1	0	1	(1)	(1)	1	1	(1)	(1)	1	0	0	(0)	(0)	0	0
2	4	1	1	0	1	(1)	(0)	0	1	(0)	(1)	0	1	(1)	(1)	1	1	1
2	4	4	1	0	1	(1)	(0)	0	1	(0)	(1)	0	1	(1)	(0)	0	1	0
2	4	6	1	0	1	(1)	(0)	0	1	(0)	(1)	0	1	(0)	(1)	0	1	0
2	4	16	1	0	1	(1)	(0)	0	1	(0)	(1)	0	0	(0)	(0)	0	0	0
2	6	1	1	0	1	(0)	(1)	0	1	(1)	(0)	0	1	(1)	(1)	1	1	1
2	6	4	1	0	1	(0)	(1)	0	1	(1)	(0)	0	1	(1)	(0)	0	1	0
2	6	6	1	0	1	(0)	(1)	0	1	(1)	(0)	0	1	(0)	(1)	0	1	0
2	6	16	1	0	1	(0)	(1)	0	1	(1)	(0)	0	0	(0)	(0)	0	0	0
2	16	1	1	0	0	(0)	(0)	0	0	(0)	(0)	0	(1)	(1)	1	1	1	1
2	16	4	1	0	0	(0)	(0)	0	0	(0)	(0)	0	(1)	(1)	0	0	0	0
2	16	6	1	0	0	(0)	(0)	0	0	(0)	(0)	0	(1)	(0)	1	0	1	0
2	16	16	1	0	0	(0)	(0)	0	0	(0)	(0)	0	(0)	(0)	0	0	0	0
4	1	1	0	0	(1)	(1)	1	1	1	(1)	(1)	1	(1)	1	(1)	(1)	1	1
4	1	4	0	0	(1)	(1)	1	1	1	(1)	(1)	1	(1)	1	(0)	(0)	1	1
4	1	6	0	0	(1)	(1)	1	1	1	(1)	(1)	1	(1)	0	(1)	(0)	0	0
4	1	16	0	0	(1)	(1)	1	1	1	(1)	(1)	1	(0)	0	(0)	(0)	0	0
4	4	1	0	0	(1)	(1)	0	0	0	(0)	(0)	0	(1)	(1)	(1)	1	1	1
4	4	4	0	0	(1)	(1)	0	0	0	(0)	(0)	0	(1)	(1)	(0)	0	0	0
4	4	6	0	0	(1)	(1)	0	0	0	(0)	(0)	0	(1)	(0)	(1)	0	0	0
4	4	16	0	0	(1)	(1)	0	0	0	(0)	(0)	0	(0)	(0)	(0)	0	0	0
4	6	1	0	0	(1)	(0)	1	0	1	(1)	(0)	0	(1)	1	(1)	1	1	1
4	6	4	0	0	(1)	(0)	1	0	1	(1)	(0)	0	(1)	1	(0)	0	1	0
4	6	6	0	0	(1)	(0)	1	0	1	(1)	(0)	0	(1)	0	(1)	0	0	0
4	6	16	0	0	(1)	(0)	1	0	1	(1)	(0)	0	(0)	0	(0)	0	0	0
4	16	1	0	0	(0)	(0)	0	0	0	(0)	(0)	0	(1)	(1)	(1)	1	1	1
4	16	4	0	0	(0)	(0)	0	0	0	(0)	(0)	0	(1)	(1)	(0)	0	0	0
4	16	6	0	0	(0)	(0)	0	0	0	(0)	(0)	0	(1)	(0)	(1)	0	0	0
4	16	16	0	0	(0)	(0)	0	0	0	(0)	(0)	0	(0)	(0)	(0)	0	0	0

We consider 4 binary variables as follows: exposure *E*, outcome *D*, confounder *C*, and selection variable *S* (see Figure 1). We show the enumeration under the assumptions of positive monotonicity of *E*, *D*, and *S* and no interaction at the individual level on the additive scale between *C* and *E* on *D* and *E* and *D* on *S* in the counterfactual framework.

^a^ Parentheses indicate that this particular outcome will never be observed.

### Four hypothetical situations

**Figure 2:**
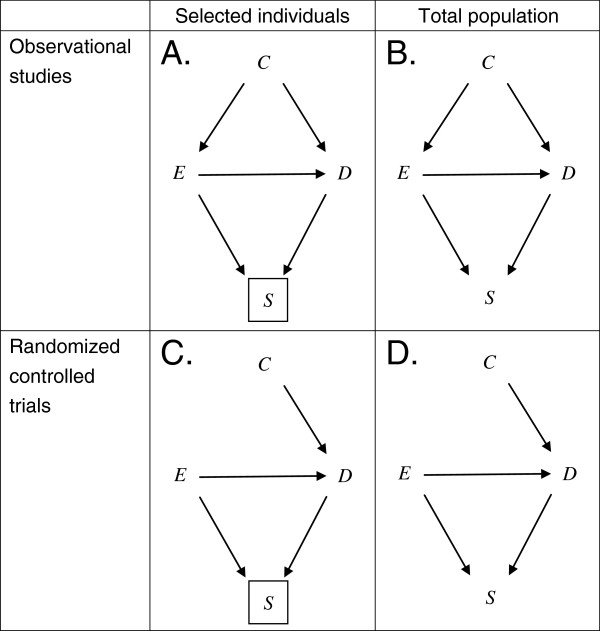
**Four causal diagrams depicting hypothetical situations.***E*, *D*, *C*, and *S* denote exposure, disease, confounder, and selection variable, respectively. *C* may act as a direct effect modifier simultaneously. The square around *S* in Figure 2A and C indicates that the analysis is restricted to those who do not drop out (i.e., *S* = 1). By contrast, 2 diagrams in Figure 2B and D show the situations in which information about total population is available to researchers. See text for details.

In Figure 2, we give an overview of 4 hypothetical situations by using DAGs. Figure 2A describes a situation in which researchers conduct an observational study and the information about a portion of subjects is unavailable due to loss to follow-up. Note that the square around *S* indicates that the analysis is restricted to those who do not drop out (i.e., *S* = 1). Investigators often encounter this situation in observational studies. Researchers should be concerned about both confounding bias and selection bias in this situation.

Subsequently, Figure 2B shows a situation in which researchers can obtain the information about the total population, including those who dropped out. In this situation, a possibility of selection bias can be ruled out since researchers do not condition on *S*.

In observational studies, researchers usually aim to eliminate confounding bias by employing some statistical procedures, e.g., standardization and inverse-probability weighting method. In other words, they aim to analytically block or remove the path between *C* and *E* by making an adequate adjustment. (Note that outcome modeling techniques such as disease risk scores focus on the path between *C* and *D*[39].) By contrast, in randomized controlled trials, researchers manipulate the value of *E* by employing certain interventions; they physically prevent *E* from varying in response to variations in *C*. Thus, as shown in Figure 2C and D, *C* would no longer have effects on *E*, and the arrow from *C* to *E* is erased or removed [14]. This should be clearly distinguished from analytic control of *C* in observational studies.

In the following sections, we demonstrate significant differences between these 4 hypothetical situations, by describing theoretical data frequencies in terms of response types.

## Results

### Describing data from observational studies in terms of response types

As demonstrated above, under the situation described in Figure 1, individuals can be classified into one of the maximum of 1,024 *EDS* response types. Despite its sophistication and usefulness, however, the response type of each individual is unobservable. Indeed, this is called a fundamental problem of causal inference [40]. Nonetheless, we can show the conceptual link between unobservable response types and observed, or observable, data frequencies in the population. In this respect, the concept of compound potential outcomes is quite useful.

**Figure 3:**
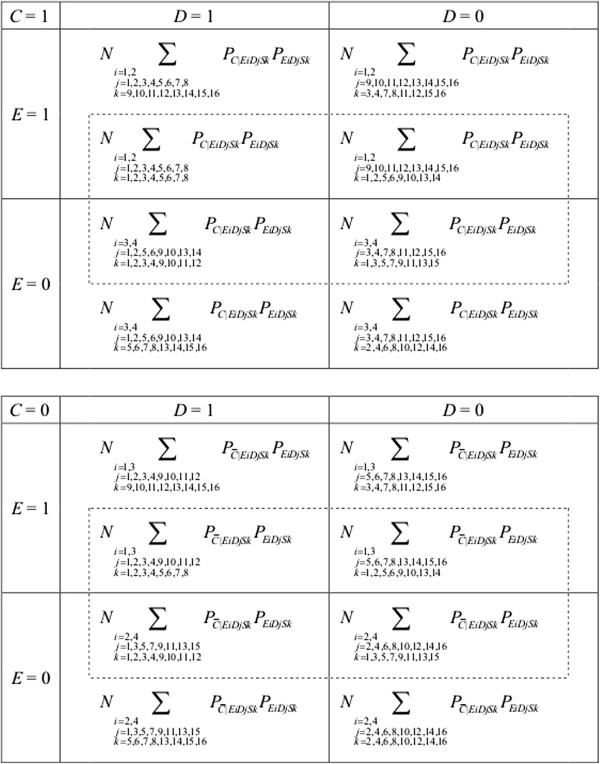
**Frequencies of individuals with 1**,**024 possible *EDS *response types in observational studies.** We consider 4 binary variables as follows: exposure *E*, outcome *D*, confounder *C*, and selection variable *S* (see Figure 1). As shown in Tables 1, 2 and 3, we consider 4 response types for *E*, 16 response types for *D*, and 16 response types for *S*. We let *EiDjSk* denote the *EDS* response type of [*E*^T^ = *i*, *D*^T^ = *j*, *S*^T^ = *k*], and let *P*_*EiDjSk*_ denote a prevalence of the individuals of *EiDjSk* response type in the total population. We also let *P*_*C*|*EiDjSk*_ and $P_{\bar{C}|\mathrm{EiDjSk}}$ denote probabilities of being exposed and unexposed to *C* among the individuals of *EiDjSk* response type, respectively. Further, *N* denotes the number of total population. Those who are classified into inner dashed rectangles represent individuals selected for analyses (i.e., *S* = 1) while those who are not classified into the rectangles represent non-selected individuals (i.e., *S* = 0). See text for details.

In Figure 3, we describe theoretical data frequencies from observational studies in terms of the 1,024 possible *EDS* response types. We let *EiDjSk* denote the *EDS* response type of [*E*^T^ = *i*, *D*^T^ = *j*, *S*^T^ = *k*] (*i* = 1, ⋯, 4, *j* = 1, ⋯, 16, *k* = 1, ⋯, 16), and let *P*_*EiDjSk*_ denote a prevalence of the individuals of *EiDjSk* response type in the total population. We also let *P*_*C*|*EiDjSk*_ and $P_{\bar{C}|\mathrm{EiDjSk}}$ denote probabilities of being exposed and unexposed to *C* among the individuals of *EiDjSk* response type, respectively. When no confusion occurs for a dichotomous variable *X*, we use the notations *X* and $\bar{X}$ in the terminologies of events of *X* = 1 and *X* = 0, respectively. For example, *C* and $\bar{C}$ mean *C* = 1 and *C* = 0, respectively. Further, *N* denotes the number of total population. Then, data frequencies in each “cell” in Figure 3 can be described either as *N* ∑ _*ijk*_*P*_*C*|*EiDjSk*_*P*_*EiDjSk*_ or $N\sum_{\mathrm{ijk}}P_{\bar{C}|\mathrm{EiDjSk}}P_{\mathrm{EiDjSk}}$. (Note that the former can be also expressed as *NP*_*C*_ ∑ _*ijk*_*P*_*EiDjSk*|*C*_ and that the latter can be expressed as $NP_{\bar{C}}\sum_{\mathrm{ijk}}P_{\mathrm{EiDjSk}|\bar{C}}$, where *P*_*C*_ and $P_{\bar{C}}$ denote probabilities of *C* and $\bar{C}$ in the total population, respectively). It should be noted that individuals can be classified into 16 “cells,” which is equivalent to a maximum possible number of 4 independent random events (i.e., *E*, *D*, *C*, and *S*). The upper and lower parts of Figure 3 show data frequencies among the subpopulation with *C* = 1 and *C* = 0, respectively. Those who are classified into inner dashed rectangles represent individuals selected for analyses (i.e., *S* = 1) while those who are not classified into the rectangles represent non-selected individuals (i.e., *S* = 0). In other words, the information about the individuals outside the rectangles is unavailable to researchers.

Notably, individuals of the same *EDS* response types can be potentially classified into 2 cells. For example, consider individual *ω* who is classified as *E*1*D*6*S*4 response type (see Table 4). This individual is, by definition, exposed to *E* = 1 irrespective of the value of *C* (i.e., *E*_1_(*ω*) = *E*_0_(*ω*) = 1). Further, individual *ω* is expected to experience outcome *D* if there had been interventions to set *C* to 1 (i.e., $D_{1E_{1}}\left(\omega \right)=D_{11}\left(\omega \right)=1$), whereas this individual is expected not to experience outcome *D* if there had been interventions to set *C* to 0 (i.e., $D_{0E_{0}}\left(\omega \right)=D_{01}\left(\omega \right)=0$). Finally, the information about this individual is, by definition, available to researchers had there been interventions to set *C* to 1 (i.e., $S_{E_{1}D_{1E_{1}}}\left(\omega \right)=S_{11}\left(\omega \right)=1$), whereas this individual is lost to follow-up had there been interventions to set *C* to 0 (i.e., $S_{E_{0}D_{0E_{0}}}\left(\omega \right)=S_{10}\left(\omega \right)=0$). Thus, in observational studies, individual *ω* of *E*1*D*6*S*4 response type can be classified into either one of the following 2 cells in Figure 3; one is *E* = 1, *D* = 1, *C* = 1, and *S* = 1 while the other is *E* = 1, *D* = 0, *C* = 0, and *S* = 0. Note that this depends on the probabilities that *C* is present or absent in individual *ω* (i.e., *P*_*C*|*E*1*D*6*S*4_ and $P_{\bar{C}|E1D6S4}$).

To summarize, Figure 3 shows theoretical data frequencies in an observational study (i.e., Figure 2A and B). The situation is, however, strikingly different when we conduct a randomized controlled trial, which will be demonstrated in the next section.

### Describing data from randomized controlled trials in terms of response types

As noted above, researchers manipulate the value of *E* in randomized controlled trials. Since researchers physically prevent *E* from varying in response to variations in *C*, we do not need to consider *E* response types when describing theoretical data frequencies in ideal randomized controlled trials; rather we focus on *D* response types and *S* response types. In other words, observed exposure status and *E* response types become independent (i.e., *E* ∐ *E*^T^) when researchers marginally intervene on *E*. Thus, theoretical data frequencies from randomized controlled trials can be described in terms of 256 (i.e., 16 × 16) possible *DS* response types, in contrast with 1,024 possible *EDS* response types.

**Figure 4:**
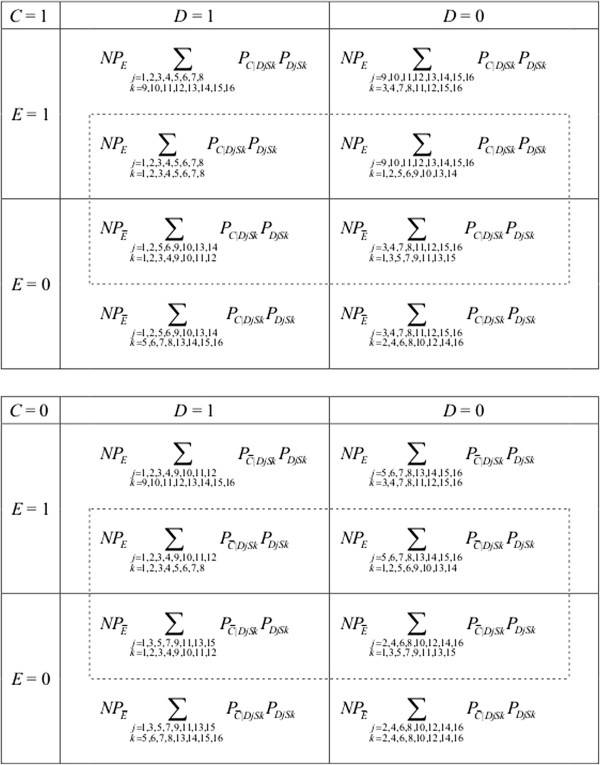
**Frequencies of individuals with 256 possible *DS *response types in randomized controlled trials.** We consider 4 binary variables as follows: exposure *E*, outcome *D*, confounder *C*, and selection variable *S* (see Figure 1). As shown in Tables 2 and 3, we consider 16 response types for *D* and 16 response types for *S*. We let *P*_*E*_ and $P_{\bar{E}}$ denote the probabilities of *E* and $\bar{E}$ in the total population, respectively. For the definition of other notations, see Figure 3. Those who are classified into inner dashed rectangles represent individuals selected for analyses (i.e., *S* = 1) while those who are not classified into the rectangles represent non-selected individuals (i.e., *S* = 0). See text for details.

We let *P*_*E*_ and $P_{\bar{E}}$ denote the probabilities of *E* and $\bar{E}$ in the total population, respectively. (For simplicity, we describe the situation of marginal randomization of *E*. However, the following discussion can be extended to the situation of stratified randomization, in which *P*_*E*_ and $P_{\bar{E}}$ may vary across the strata of *C*.) Figure 4 shows distributions of individuals of the 256 possible *DS* response types in a randomized controlled trial. Note that data frequencies in each “cell” in Figure 4 can be described as *NP*_*E*_ ∑ _*jk*_*P*_*C*|*DjSk*_*P*_*DjSk*_, $NP_{\bar{E}}\sum_{\mathrm{jk}}P_{C|\mathrm{DjSk}}P_{\mathrm{DjSk}}$, $NP_{E}\sum_{\mathrm{jk}}P_{\bar{C}|\mathrm{DjSk}}P_{\mathrm{DjSk}}$, or $NP_{\bar{E}}\sum_{\mathrm{jk}}P_{\bar{C}|\mathrm{DjSk}}P_{\mathrm{DjSk}}$. (Note that these can be also expressed as *NP*_*E*_*P*_*C*_ ∑ _*jk*_*P*_*DjSk*|*C*_, $NP_{\bar{E}}P_{C}\sum_{\mathrm{jk}}P_{\mathrm{DjSk}|C}$, $NP_{E}P_{\bar{C}}\sum_{\mathrm{jk}}P_{\mathrm{DjSk}|\bar{C}}$, and $NP_{\bar{E}}P_{\bar{C}}\sum_{\mathrm{jk}}P_{\mathrm{DjSk}|\bar{C}}$, respectively.) As in the case of observational studies (Figure 3), individuals can be classified into 16 “cells” in Figure 4.

The theoretical data frequencies in Figure 4 can be explained as a re-distribution of individuals in Figure 3. For example, consider individual *ω* who is classified as either *E*1*D*6*S*4 response type or *E*2*D*6*S*4 response type. In observational studies, if the value of *C* of individual *ω* is 1, this individual is classified into an upper-left cell within the inner dashed rectangle of the upper part of Figure 3, i.e., *E* = 1, *D* = 1, *C* = 1, and *S* = 1. Then, when this individual is forced to be exposed to *E* in a randomized controlled trial, this individual would remain in the upper-left cell within the inner dashed rectangle of the upper part of Figure 4. Note that neither *D* response types nor *S* response types of this individual change by the intervention on *E*. By contrast, if individual *ω* is forced to be unexposed to *E*, this individual “moves” to a lower-left cell within the inner dashed rectangle of the upper part of Figure 4, i.e., *E* = 0, *D* = 1, *C* = 1, and *S* = 1. On the other hand, consider individual *ω* who is classified as either *E*3*D*6*S*4 response type or *E*4*D*6*S*4 response type. In observational studies, if the value of *C* of individual *ω* is 1, this individual is classified into a lower-left cell within the inner dashed rectangle of the upper part of Figure 3, i.e., *E* = 0, *D* = 1, *C* = 1, and *S* = 1. Then, in randomized controlled trials, if this individual is forced to be unexposed to *E*, this individual would remain in the lower-left cell within the inner dashed rectangle of the upper part of Figure 4. Meanwhile, if this individual is forced to be exposed to *E*, this individual “moves” to an upper-left cell within the inner dashed rectangle of the upper part of Figure 4, i.e., *E* = 1, *D* = 1, *C* = 1, and *S* = 1. These re-distributions can be summarized as

(3) $$\begin{array}{l} P_{E}\left(N\sum_{i=1,2}P_{C|\mathrm{EiD}6S4}P_{\mathrm{EiD}6S4}+N\sum_{i=3,4}P_{C|\mathrm{EiD}6S4}P_{\mathrm{EiD}6S4}\right)\\ \;=NP_{E}\sum_{i=1,2,3,4}P_{C}P_{\mathrm{EiD}6S4|C}\\ \;=NP_{E}P_{C}P_{D6S4|C}\\ \;=NP_{E}P_{C|D6S4}P_{D6S4}, \end{array}$$

and

(4) $$\begin{array}{l} P_{\bar{E}}\left(N\sum_{i=1,2}P_{C|\mathrm{EiD}6S4}P_{\mathrm{EiD}6S4}+N\sum_{i=3,4}P_{C|\mathrm{EiD}6S4}P_{\mathrm{EiD}6S4}\right)\\ \;=NP_{\bar{E}}\sum_{i=1,2,3,4}P_{C}P_{\mathrm{EiD}6S4|C}\\ \;=NP_{\bar{E}}P_{C}P_{D6S4|C}\\ \;=NP_{\bar{E}}P_{C|D6S4}P_{D6S4}. \end{array}$$

Note that the numbers in the parentheses of left-hand sides of equations 3 and 4 are based on the subpopulation of *C* = 1 in observational studies (i.e., upper part of Figure 3), whereas the right-hand sides of these equations are based on the subpopulation of *C* = 1 in randomized controlled trials (i.e., upper part of Figure 4). In other words, these equations explain how individuals of subpopulation of *C* = 1 are re-distributed as a result of intervention on *E*.

Analogous discussion applies when the value of *C* is 0 among the individuals of *E*1*D*6*S*4, *E*2*D*6*S*4, *E*3*D*6*S*4, or *E*4*D*6*S*4 response types. Note that, in observational studies, these individuals are classified in either an upper-right cell (i.e., *E* = 1, *D* = 0, *C* = 0, and *S* = 0) or a lower-right cell (i.e., *E* = 0, *D* = 0, *C* = 0, and *S* = 0) outside the inner dashed rectangle of the lower part of Figure 3. The re-distributions of these individuals as a result of intervention on *E* can be summarized as

(5) $$\begin{array}{l} P_{E}\left(N\sum_{i=1,3}P_{\bar{C}|\mathrm{EiD}6S4}P_{\mathrm{EiD}6S4}+N\sum_{i=2,4}P_{\bar{C}|\mathrm{EiD}6S4}P_{\mathrm{EiD}6S4}\right)\\ \;=NP_{E}\sum_{i=1,2,3,4}P_{\bar{C}}P_{\mathrm{EiD}6S4|\bar{C}}\\ \;=NP_{E}P_{\bar{C}}P_{D6S4|\bar{C}}\\ \;=NP_{E}P_{\bar{C}|D6S4}P_{D6S4},\; \end{array}$$

and

(6) $$\begin{array}{l} P_{\bar{E}}\left(N\sum_{i=1,3}P_{\bar{C}|\mathrm{EiD}6S4}P_{\mathrm{EiD}6S4}+N\sum_{i=2,4}P_{\bar{C}|\mathrm{EiD}6S4}P_{\mathrm{EiD}6S4}\right)\\ \;=NP_{\bar{E}}\sum_{i=1,2,3,4}P_{\bar{C}}P_{\mathrm{EiD}6S4|\bar{C}}\\ \;=NP_{\bar{E}}P_{\bar{C}}P_{D6S4|\bar{C}}\\ \;=NP_{\bar{E}}P_{\bar{C}|D6S4}P_{D6S4}. \end{array}$$

Again, the numbers in the parentheses of left-hand sides of equations 5 and 6 are based on the subpopulation of *C* = 0 in observational studies (i.e., lower part of Figure 3), whereas the right-hand sides of these equations are based on the subpopulation of *C* = 0 in randomized controlled trials (i.e., lower part of Figure 4). In other words, these equations explain how individuals of subpopulation of *C* = 0 are re-distributed as a result of intervention on *E*. It should be noted that these re-distributions do not occur across the upper and lower parts of Figures 3 and 4 because *C* precedes *E* temporally and the value of *C* is, by definition, predetermined before intervention on *E*. These discussions also demonstrate that, in Figure 4, individuals of the same *DS* response types can be potentially classified into 4 cells, depending on the probability of being exposed or unexposed to *C* (i.e., *P*_*C*|*DjSk*_ or $P_{\bar{C}|\mathrm{DjSk}}$) and the probability of being exposed or unexposed to *E* (i.e., *P*_*E*_ or $P_{\bar{E}}$).

Note that, when the information about the total population is available, both marginal and conditional exchangeability assumptions are met in Figure 4; the distributions of *DS* response types are comparable between the exposed and unexposed groups. However, when the information about those who dropped out is not available, exchangeability assumptions do not hold, either conditionally or unconditionally. See (Additional file 1: Appendix 1) for a discussion of positivity – another fundamental assumption for causal inference [41-43].

### Epidemiologic measures in terms of response types

The descriptions of data frequencies in Figures 3 and 4 have a crucial implication, demonstrating significant distinctions between association measures and effect measures [9]. In the following sections, we continue to focus our discussion on RRs, which can be extended to other measures. Note also that, although epidemiologic measures can be defined for a variety of target population (e.g., the exposed and the unexposed), the following discussion focuses on the situation in which target population is the total population. Furthermore, we also discuss epidemiologic measures in the subpopulation defined by *C* or *S*.

In observational studies (Figure 2A and B), researchers can readily calculate associational RRs by referring to the notations in Figure 3. In particular, when no information is available about those who dropped out (Figure 2A), one can calculate an associational RR_*S*=1_ by using the information about individuals classified into the inner rectangles in Figure 3. Then, as shown in (Additional file 2: Table S1), associational RR_*S*=1_ can be described in terms of a probability of being exposed or unexposed to *C* among the individuals of *EiDjSk* response type (i.e., *P*_*C*|*EiDjSk*_ or $P_{\bar{C}|\mathrm{EiDjSk}}$) and a prevalence of the individuals of *EiDjSk* response type in the total population (i.e., *P*_*EiDjSk*_) (equation A1). Meanwhile, when researchers are capable of gathering information about those who dropped out (Figure 2B), the information about individuals of *S* response types 1 through 16 is available, which yields an associational RR (equation A4).

By contrast, when researchers obtain data from randomized controlled trials (Figure 2C and D), their frequencies can be described in a different way, as shown in Figure 4. In these cases, researchers can calculate causal RRs to infer causality between *E* and *D*. When no information is available about those who dropped out (Figure 2C), one can calculate a causal RR_*S*=1_ by using the information about individuals classified into the inner rectangles in Figure 4. Then, as shown in (Additional file 2: Table S2), causal RR_*S*=1_ can be described in terms of a probability of being exposed or unexposed to *C* among the individuals of *DjSk* response type (i.e., *P*_*C*|*DjSk*_ or $P_{\bar{C}|\mathrm{DjSk}}$) and a prevalence of the individuals of *DjSk* response type in the total population (i.e., *P*_*DjSk*_) (equation A7). In ideal randomized controlled trials without loss to follow-up (Figure 2D), the information about individuals of *S* response types 1 through 16 is available, which yields a causal RR (equation A10). We should note that the causal RR shown in equation A10 is an alternative notation of the causal RR shown in equation 1 in terms of response types (see Additional file 1: Appendix 2).

Note that, even in ideal (either marginal or stratified) randomized controlled trials, one may observe a heterogeneity between stratum-specific causal RRs, which will be addressed in the section entitled “Modification of epidemiologic measures”.

### Confounding bias

In this section, we aim to further clarify the concept of confounding bias in the counterfactual framework, by describing it in terms of response types.

We show a sufficient condition to estimate effect measures in observational studies by adjusting for confounding bias in terms of response types. In this case, we use effect measures in the total population in ideal randomized controlled trials (i.e., causal RR) as a gold standard. As noted above, confounding bias is induced by a common cause *C* of *E* and *D*. Thus, to show a sufficient condition to adjust for confounding bias, we need to compare association measures in the total population in observational studies (Figure 2B) and effect measures in the total population in randomized controlled trials (Figure 2D) In other words, a sufficient condition to adjust for confounding bias can be described as: adjusted associational RR = causal RR. Note that we here compare 2 distinct types of epidemiologic measures, which are obtained from distinct study designs.

To adjust for confounding bias in observational studies, one may calculate a weighted average of stratum-specific associational RRs, or standardization, expecting to estimate a causal RR. By using stratum-specific associational RRs (equations A5 and A6), this procedure can be described in terms of response types as follows:

(7) $$\begin{array}{l} \frac{P_{C}\left(\frac{\sum_{\begin{array}{l} i=1,2\\ j=1,2,3,4,5,6,7,8 \end{array}}P_{C|\mathrm{EiDj}}P_{\mathrm{EiDj}}}{\sum_{i=1,2}P_{C|\mathrm{Ei}}P_{\mathrm{Ei}}}\right)+P_{\bar{C}}\left(\frac{\sum_{\begin{array}{l} i=1,3\\ j=1,2,3,4,9,10,11,12 \end{array}}P_{\bar{C}|\mathrm{EiDj}}P_{\mathrm{EiDj}}}{\sum_{i=1,3}P_{\bar{C}|\mathrm{Ei}}P_{\mathrm{Ei}}}\right)}{P_{C}\left(\frac{\sum_{\begin{array}{l} i=3,4\\ j=1,2,5,6,9,10,13,14 \end{array}}P_{C|\mathrm{EiDj}}P_{\mathrm{EiDj}}}{\sum_{i=3,4}P_{C|\mathrm{Ei}}P_{\mathrm{Ei}}}\right)+P_{\bar{C}}\left(\frac{\sum_{\begin{array}{l} i=2,4\\ j=1,3,5,9,11,13,15 \end{array}}P_{\bar{C}|\mathrm{EiDj}}P_{\mathrm{EiDj}}}{\sum_{i=2,4}P_{\bar{C}|\mathrm{Ei}}P_{\mathrm{Ei}}}\right)}\\ =\frac{\left(\frac{\sum_{\begin{array}{l} i=1,2\\ j=1,2,3,4,5,6,7,8 \end{array}}P_{C}P_{\mathrm{EiDj}|C}}{\sum_{i=1,2}P_{\mathrm{Ei}|C}}+\frac{\sum_{\begin{array}{l} i=1,3\\ j=1,2,3,4,9,10,11,12 \end{array}}P_{\bar{C}}P_{\mathrm{EiDj}|\bar{C}}}{\sum_{i=1,3}P_{\mathrm{Ei}|\bar{C}}}\right)}{\left(\frac{\sum_{\begin{array}{l} i=3,4\\ j=1,2,5,6,9,10,13,14 \end{array}}P_{C}P_{\mathrm{EiDj}|C}}{\sum_{i=3,4}P_{\mathrm{Ei}|C}}+\frac{\sum_{\begin{array}{l} i=2,4\\ j=1,3,5,9,11,13,15 \end{array}}P_{\bar{C}}P_{\mathrm{EiDj}|\bar{C}}}{\sum_{i=2,4}P_{\mathrm{Ei}|\bar{C}}}\right)}. \end{array}$$

Notably, this is not equivalent to a causal RR (equation A10). In other words, this stratification-based procedure does not “delete” the arrow from *C* to *E* in Figure 2B, yielding subtly different measures from effect measures. When one can assume conditional exchangeability (i.e., *E*∐*D*_*e*_|*C* for ∀*e*), the weighted average of stratum-specific associational RR can be expressed as

(8) $$\frac{\sum_{j=1,2,3,4,5,6,7,8}P_{C|\mathrm{Dj}}P_{\mathrm{Dj}}+\sum_{j=1,2,3,4,9,10,11,12}P_{\bar{C}|\mathrm{Dj}}P_{\mathrm{Dj}}}{\sum_{j=1,2,5,6,9,10,13,14}P_{C|\mathrm{Dj}}P_{\mathrm{Dj}}+\sum_{j=1,3,5,9,11,13,15}P_{\bar{C}|\mathrm{Dj}}P_{\mathrm{Dj}}},$$

which is equivalent to a causal RR in equation A10 (see Additional file 1: Appendix 3). Indeed, the condition *E*∐*D*_*e*_|*C* for ∀*e* is a sufficient condition to estimate effect measures in observational studies by adjusting for confounding bias, and the assumption of exchangeability often gets most of the attention in discussions about causal inference [44]. Unfortunately, however, the condition is not guaranteed in observational studies, and expert knowledge is required. Importantly, the assumption of conditional exchangeability, i.e., *E*∐*D*_*e*_|*C* for ∀*e*, is subtly weaker than another sufficient condition to estimate effect measures in observational studies, i.e., full conditional exchangeability, or *E*∐(*D*_*e*=1_, *D*_*e*=0_)|*C*[45]. It may be difficult, however, to imagine a practical scenario where the former holds but not the latter [46], and the word “exchangeability” has been sometimes used interchangeably in the literature. (A combination of full exchangeability and positivity has been termed “strongly ignorable treatment assignment” assumption or “strong ignorability,” whereas a combination of exchangeability and positivity has been termed “weakly ignorable treatment assignment” assumption or “weak ignorability” [2,47,48].) By comparing equations 7 and 8, we can show that the conditions *E*^T^ ∐ *D*^T^|*C* and *E* ∐ *D*^T^|*C* are also sufficient conditions to estimate effect measures in observational studies by adjusting for confounding bias (see Additional file 1: Appendix 3). In Additional file 1: Appendix 4, we show a proof of the following inclusion relation:

$$E^{T}∐D^{T}|C\Rightarrow E∐D^{T}|C\Rightarrow E∐\left(D_{e=1},D_{e=0}\right)|C\Rightarrow E∐D_{e}|C\;\mathrm{for}\;\forall e$$

The subtle differences between *E*^T^ ∐ *D*^T^|*C* and *E* ∐ *D*^T^|*C* are described graphically in the section entitled “Extended causal diagrams integrating response types”. It is worthwhile to mention that the condition *E*^T^ ∐ *D*^T^|*C* is not guaranteed in randomized controlled trials.

The above discussion implies that analytic adjustment for *C* in observational studies has consequences quite different from those of physical control in randomized controlled trials. Even when adequate analytic control of *C* may be envisaged in observational studies, researchers cannot estimate effect measures without the assumption external to data. See Additional file 1: Appendix 5 for a discussion of recently-introduced assumptions to compensate for a lack of randomization.

### Selection bias

In this section, we aim to further clarify the concept of selection bias in the counterfactual framework, by describing it in terms of response types.

We show sufficient conditions for non-selection bias in terms of response types. As explained above, selection bias is induced by conditioning on a common effect of *E* and *D* (Figure 2A and C). Thus, to show sufficient conditions for non-selection bias, we need to specify epidemiologic measures, i.e., association measures or effect measures. With regard to association measures, a sufficient condition for non-selection bias is described as associational RR_*S*=1_ = associational RR (see equations A1 and A4). Likewise, a sufficient condition for non-selection bias for effect measures is described as causal RR_*S*=1_ = causal RR (see equations A7 and A10). It is worthwhile to mention that, when discussing selection bias, one need to specify a stratum of *S*[21]. In most cases, researchers are interested in the presence and the degree of selection bias among the subjects who do not drop out. Thus, we here show sufficient conditions for non-selection bias in a stratum *S* = 1. As explained later by using extended causal diagrams, selection bias results in violation of *E* ∐ *D*^T^ even when exposure is randomly assigned.

### Modification of epidemiologic measures

For decades, epidemiologists have used the term “effect modification” in a broad context, simply referring to a variation in the selected effect measure for the factor under study across levels of another factor [49]. In this respect, a recent paper clarified the distinction between interaction and effect modification within the counterfactual framework [31]. It is also well known that the presence, direction, and size of modification can be dependent on the choice of measure [50]. Since the term “effect modification” is ambiguous, it is now recommended to specify the measures more precisely, e.g., risk-difference modification [50]. The above discussion implies that researchers need to distinguish association-measure modification and effect-measure modification. For example, when the information about total population is available in a randomized controlled trial, causal-RR modification is defined to be present if stratum-specific causal RRs from each subpopulation varies across the strata of *C*, i.e., causal RR_*C*=1_ ≠ causal RR_*C*=0_ (see equations A11 and A12). When stratum-specific causal RRs are (approximately) homogeneous or uniform across strata, researchers usually pool the data to calculate a causal RR in the total population (i.e., causal RR). In a similar manner, one can define associational-RR modification (see equations A5 and A6). Only if it is appropriate to pool the data across the strata of *C*, one can validly interpret associational RRs in the total population.

Notably, the presence of association-measure modification does not necessarily imply the presence of effect-measure modification, and vice versa.

### Extended causal diagrams integrating response types

In this section, we attempt to explain the concept of bias by extending causal diagrams, which integrate response types and observed variables. Although these causal diagrams, or extended DAGs, may appear less intuitive, they maintain the integrity of the original DAGs and would be of great use in graphically describing the findings discussed in this study. In particular, by integrating response types and observed variables, we can readily understand subtle differences between *E*^T^ ∐ *D*^T^|*C* and *E* ∐ *D*^T^|*C*, demonstrating sufficient conditions to estimate effect measures in observational studies.

**Figure 5:**
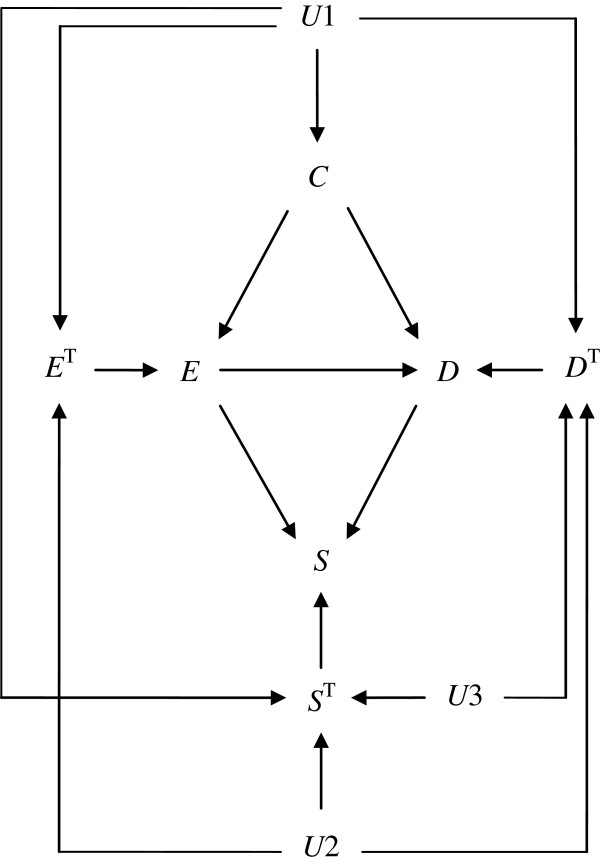
An extended causal diagram depicting a hypothetical example.

Figure 5 shows the hypothetical situation described in Figure 1 by integrating response types of *E*, *D*, and *S* (i.e., *E*^T^, *D*^T^, and *S*^T^, respectively). First, note that the only arrows emanating from the response types point to the corresponding observed variables, i.e., *E*^T^ → *E*, *D*^T^ → *D*, and *S*^T^ → *S*. Then, to describe the underlying correlation between *E*^T^, *D*^T^, and *S*^T^, we use a total of 3 unmeasured common causes, *U*1, *U*2, and *U*3, which are independent of each other. In other words, these unmeasured common causes as a whole represent underlying personal characteristics determining his/her response types of *E*, *D*, and *S*. (Note that, unlike *U*1, *U*2, and *U*3, we assume that *C* does not determine his/her response types. In other words, we assume that *U*1, *U*2, and *U*3 precede response types, while *C* does not.) It would be worth to mention that Figure 5 well describes how the observed variables are determined in response to the corresponding response types and their measured parent(s). For example, each individual has 2 potential outcomes of *E*: the outcome that would occur if *C* is present in that individual, *E*_1_, and the outcome that would occur if *C* is absent in that individual, *E*_0_. Thus, we observe *E* = *CE*_1_ + (1 − *C*)*E*_0_. In Figure 5, this equation is illustrated by the only 2 arrows from *E*^T^ and *C* to *E*.

**Figure 6:**
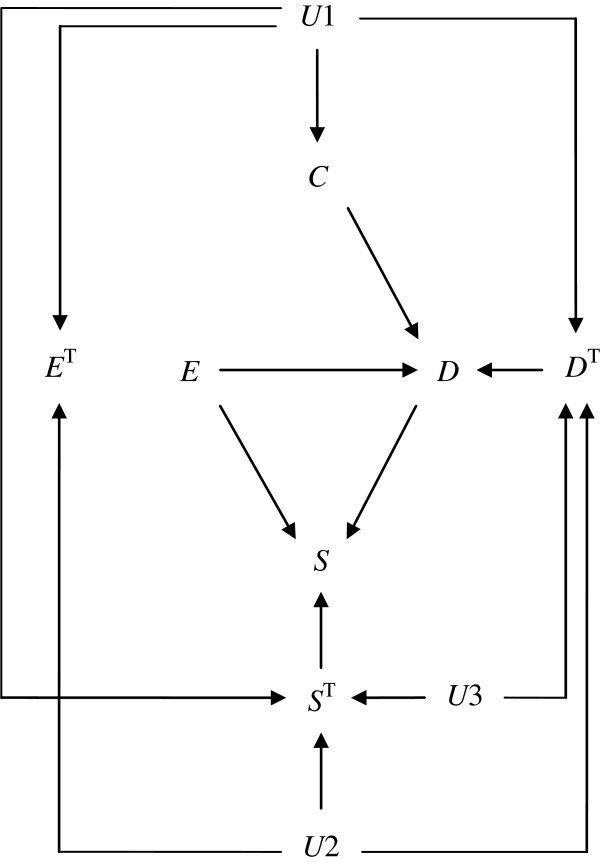
An extended causal diagram depicting a hypothetical situation under marginal randomization.

In randomized controlled trials, in which *E* is marginally randomized, researchers physically prevent *E* from varying in response to variations in *C* by intervening on *E*. Thus, by applying the rule used in the standard DAG theory, the 2 arrows pointing to *E* in Figure 5 are removed. As a result, observed exposure status and *E* response types become independent (i.e., *E* ∐ *E*^T^), as shown in Figure 6. In addition, observed exposure status becomes independent of *D* response types and *S* response types (i.e., $E∐D^{T}$ and $E∐S^{T}$, respectively) because the value of *E* is, by definition, determined randomly. Note that $E∐D^{T}$ implies an assumption of (full) exchangeability. Trivially, observed exposure status is also independent of *D*^T^ given *C*, i.e., $E∐D^{T}\left|C\right)$, thus implying the assumption of (full) conditional exchangeability. Importantly, even when adjusting for *C*, marginal randomization of *E* does not result in independence between *E* response types and *D* response types due to 2 open paths, *E*^T^ ← *U*1 → *D*^T^ and *E*^T^ ← *U*2 → *D*^T^. If we adjust for *U*1 and *U*2, they become independent. Finally, Figure 6 also clearly shows that selection bias results in violation of $E∐D^{T}$; when some of the subjects are lost to follow up, 7 marginally blocked paths between *E* and *D*^T^ (i.e., *E* → *S* ← *D* ← *D*^T^, *E* → *S* ← *S*^T^ ← *U*1 → *D*^T^, *E* → *S* ← *S*^T^ ← *U*2 → *D*^T^, *E* → *S* ← *S*^T^ ← *U*3 → *D*^T^, *E* → *D* → *S* ← *S*^T^ ← *U*1 → *D*^T^, *E* → *D* → *S* ← *S*^T^ ← *U*2 → *D*^T^, and *E* → *D* → *S* ← *S*^T^ ← *U*3 → *D*^T^) become open because we condition on the collider *S*. Indeed, extended DAGs are of great use to demonstrate that both confounding bias and selection bias result in lack of (full) exchangeability of the exposed and unexposed groups.

**Figure 7:**
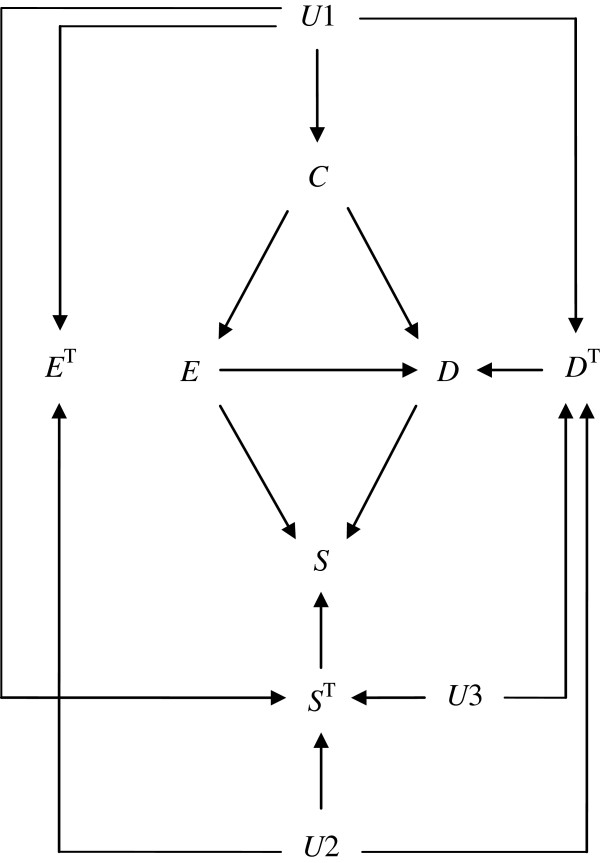
An extended causal diagram depicting a hypothetical situation under stratified randomization.

Meanwhile, when using stratified randomization of *E* by *C*, researchers physically prevent *E* from varying in response to variations in *E* response types, but the probability of *E* may vary across the strata of *C*. Thus, unlike the marginal randomization of *E*, of the 2 arrows pointing to *E* in Figure 5, only the arrow from *E*^T^ to *E* is removed (Figure 7). As a result, there is an open path between *E* and *E*^T^, i.e., *E* ← *C* ← *U*1 → *E*^T^, which can be blocked by adjusting for *C* (i.e., $E∐E^{T}\left|C\right)$). Further, although there is an open path between *E* and *D*^T^, i.e., *E* ← *C* ← *U*1 → *D*^T^, this path can be blocked by adjusting for *C* (i.e., $E∐D^{T}\left|C\right)$), which implies that the assumption of (full) conditional exchangeability can be readily met in stratified randomization of *E*. Finally, there is an open path between *E* and *S*^T^, i.e., *E* ← *C* ← *U*1 → *S*^T^, which can be also blocked by adjusting for *C* (i.e., $E∐S^{T}\left|C\right)$). Note that, like Figure 6, even when adjusting for *C*, stratified randomization of *E* does not result in independence between *E* response types and *D* response types, and we need to additionally adjust for *U*1 and *U*2.

**Figure 8:**
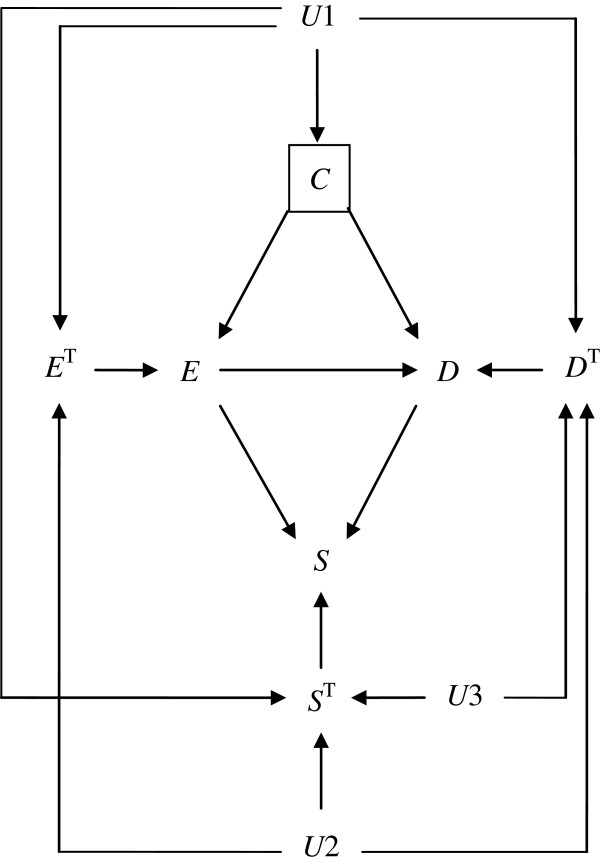
An extended causal diagram depicting a hypothetical situation in observational studies.

Figure 8 shows the situation in observational studies, in which researchers stratify by *C* to calculate a weighted average of stratum-specific association measures. (A square around *C* means that we condition on *C*.) Unlike marginal or stratified randomization of *E*, observed exposure status is determined in response to *E* response types as well as the status of *C*. Therefore, no arrows pointing to *E* are removed in Figure 8. Note that, in Figure 8, *E* and *D*^T^ would be marginally connected via the following 3 paths, i.e., *E* ← *E*^T^ ← *U*1 → *D*^T^, *E* ← *E*^T^ ← *U*2 → *D*^T^, and *E* ← *C* ← *U*1 → *D*^T^. When we condition on *C*, only the third path can be blocked, and *E* and *D*^T^ remain connected via the first 2 paths. Notably, the 3 paths can be theoretically blocked by conditioning on *U*1 and *U*2. In other words, a sufficient condition of $E∐D^{T}\left|C\right)$ is to adjust for *U*1 and *U*2 in observational studies. Meanwhile, in equation 8, we demonstrate that the condition $E^{T}∐D^{T}\left|C\right)$ is a sufficient condition to estimate effect measures in observational studies without loss to follow-up. Indeed, *E*^T^ and *D*^T^ are not independent given *C* in Figure 8, and they are connected via the following 2 paths, conditional on *C*, i.e., *E*^T^ ← *U*1 → *D*^T^ and *E*^T^ ← *U*2 → *D*^T^. Note that both paths can be theoretically blocked by conditioning on *U*1 and *U*2. To summarize, although $E^{T}∐D^{T}\left|C\right)$ and $E∐D^{T}\left|C\right)$ are sufficient conditions to estimate effect measures in observational studies, neither is guaranteed in observational studies, and expert knowledge is required. In particular, although $E^{T}∐D^{T}\left|C\right)$ is stronger than $E∐D^{T}\left|C\right)$, we need to adjust for *U*1 and *U*2 to achieve either condition as shown in Figure 8.

Finally, it is worthwhile to mention that the perspectives of the extended DAGs are different from those of the twin network method, which has been developed to deal with counterfactual values in DAGs [2]. This graphical method uses two networks, one to represent the actual world and one to represent the hypothetical world. Thus, this method is used to represent the causal relations under intervention. The aim of our extended DAGs is to integrate response types and observed variables, which is thus applicable to observational studies as well as randomized controlled trials. As a consequence, we can use the extended DAGs to describe the sufficient conditions to infer causality in observational studies in terms of response types.

## Discussion

We have clarified the concepts of bias and effect modification in the counterfactual framework, by describing theoretical data frequencies from observational studies and randomized controlled trials in terms of response types. Although these concepts have been extensively explained in the epidemiologic literature, most of the studies have discussed them separately. In this article, we have highlighted the relations between these concepts, by discussing them simultaneously. The present findings would somehow clarify the link between the assumptions for making causal inferences in observational studies and the counterfactual approach, demonstrating the inherent distinctions between observational studies and randomized controlled trials. The extension of DAGs using response types maintains the integrity of the original DAGs, which allows one to understand the underlying causal structure discussed in this study.

We have shown a hitherto unrecognized sufficient condition $E^{T}∐D^{T}\left|C\right)$ to estimate effect measures in observational studies by adjusting for confounding bias. This condition is stronger than the assumption of (full) conditional exchangeability, and it is not straightforward to discuss technical advantages of the hitherto unrecognized condition. Such consideration however would enable one to further understand the conceptual link between unobservable response types and observed, or observable, data frequencies in the population. This would also facilitate understanding of the underlying causal structures of bias and effect modification.

In this article, we use a simple hypothetical situation, including only 4 binary variables. Thus, it should be noted that the present study does not encompass more complicated situations, e.g., M-bias [51]. It is however worthwhile to mention that the condition $E^{T}∐D^{T}\left|C\right)$ is applicable even when an exposure and an outcome are polytomous variables, because our discussion based on the extended DAGs does not restrict the type of variables. When considering situations in which there are some confounders, the present finding would apply by defining and estimating a function of measured confounders that can be treated as a single confounder. It should be also noted that we focused only on direct effect modification, and thus, the present discussion does not necessarily apply to other types of effect modification, i.e., indirect effect modification, effect modification by proxy, and effect modification by a common cause [22]. Further, this study does not address the issue of information bias or measurement error. Recent studies have discussed how DAGs can be used to represent them [52-55], which should be addressed further in future studies.

## Conclusion

As shown in the present study, researchers should recognize inherent limitations of observational studies in estimating causal effects. It should be emphasized, however, that the recognition should come in the interpretation of the evidence when trying to draw conclusions, not in the statement of research goals or study design and conduct phases [56]. The data from observational studies yield measures of association and those who examine the data should strive to impose a meaning based on their expert knowledge on each occasion, which would improve causal interpretations.

## Abbreviations

DAG: Directed acyclic graph; RR: Risk ratio

## Competing interests

The authors declare that they have no competing interests.

## Authors’ contributions

ES conceptualized the authors' views and drafted the manuscript. TM, TT, and EY critically revised the manuscript for intellectual content. EY supervised the study. All authors read and approved the final manuscript.

## Authors’ information

ES is Assistant Professor of Epidemiology at Okayama University. His primary research interest concerns improving causal interpretations of observational studies. TM was a Research Fellow of Epidemiology when this study was conducted. He is currently working as Assistant Professor in Center for Innovative Clinical Medicine at Okayama University Hospital. TT, as a Professor of Environmental Epidemiology, has evaluated a variety of health effects of environmental factors to advance the public’s health. EY, as a Professor of Statistics, is interested in contributing to the advancement of statistical theories necessary for causal inference.

## Pre-publication history

The pre-publication history for this paper can be accessed here:

http://www.biomedcentral.com/1471-2288/13/101/prepub

## Supplementary Material

Additional file 1 Appendices 1 to 5. Click here for file

Additional file 2: Tables S1 and S2 Risk ratios in terms of response types. Click here for file
