# Supplementary material for: A counterfactual approach to bias and effect modification in terms of response types
Source: BMC Med Res Methodol. 2013 Jul 31;13:101. doi: 10.1186/1471-2288-13-101 (PMC3765813; doi:10.1186/1471-2288-13-101)
Supplement: Additional file 1 — Appendices 1 to 5. [file 1471-2288-13-101-S1.pdf]

A counterfactual approach to bias and effect modification in terms of response types

Etsuji Suzuki, Toshiharu Mitsuhashi, Toshihide Tsuda, and Eiji Yamamoto

Additional file 1

Appendices 1 to 5

Appendix 1: positivity condition

In addition to exchangeability, recent studies have emphasized the significance of positivity condition, sometimes referred to as the experimental treatment assignment assumption, to infer causation [1-3]. Positivity means that we must ensure that there is a nonzero probability of being assigned to each of the treatment levels at every combination of the values of the observed confounder(s) in the population under study. By using the notation in the present study, positivity is described as: if $P[C=c]>0$ then $P[E=e \mid C=c]>0$ for $\forall\, e$. In (either marginally or stratified) randomized controlled trials, positivity is taken for granted. In observational studies, however, positivity is not guaranteed.

When the information about those who did not drop out is available in observational studies (Figure 2A), positivity assumption can be described in terms of *EDS* response types as follows (see Figure 3): If $P[C=1]>0$ then

$$P\left[E=1 \mid C=1\right]>0 \Leftrightarrow \sum_{\substack{i=1,2 \\ j=1,2,3,4,5,6,7,8 \\ k=1,2,3,4,5,6,7,8}} P_{C|EiDjSk} P_{EiDjSk} + \sum_{\substack{i=1,2 \\ j=9,10,11,12,13,14,15,16 \\ k=1,2,5,6,9,10,13,14}} P_{C|EiDjSk} P_{EiDjSk} > 0,$$

and

$$P\left[E=0 \mid C=1\right]>0 \Leftrightarrow \sum_{\substack{i=3,4 \\ j=1,2,5,6,9,10,13,14 \\ k=1,2,3,4,9,10,11,12}} P_{C|EiDjSk} P_{EiDjSk} + \sum_{\substack{i=3,4 \\ j=3,4,7,8,11,12,15,16 \\ k=1,3,5,7,9,11,13,15}} P_{C|EiDjSk} P_{EiDjSk} > 0.$$

In addition, if $P[C=0]>0$ then

$$P\left[E=1 \mid C=0\right]>0 \Leftrightarrow \sum_{\substack{i=1,3 \\ j=1,2,3,4,9,10,11,12 \\ k=1,2,3,4,5,6,7,8}} P_{\bar{C}|EiDjSk} P_{EiDjSk} + \sum_{\substack{i=1,3 \\ j=5,6,7,8,13,14,15,16 \\ k=1,2,5,6,9,10,13,14}} P_{\bar{C}|EiDjSk} P_{EiDjSk} > 0,$$

and

$$P\left[E=0 \mid C=0\right]>0 \Leftrightarrow \sum_{\substack{i=2,4 \\ j=1,3,5,7,9,11,13,15 \\ k=1,2,3,4,9,10,11,12}} P_{\bar{C}|EiDjSk} P_{EiDjSk} + \sum_{\substack{i=2,4 \\ j=2,4,6,8,10,12,14,16 \\ k=1,3,5,7,9,11,13,15}} P_{\bar{C}|EiDjSk} P_{EiDjSk} > 0.$$

Thus, even under the assumption that there are no *E* response types 2 or 3 in the population under study (i.e., $E^{\mathrm{T}}(\omega)=1,4$ for $\forall\omega$), positivity can be met. Analogously, even under the assumption that there are no *E* response types 1 or 4 in the population under study (i.e., $E^{\mathrm{T}}(\omega)=2,3$ for $\forall\omega$), positivity can be also met. By contrast, under the assumptions of either $E^{\mathrm{T}}(\omega)=1,2$ for $\forall\omega$, $E^{\mathrm{T}}(\omega)=1,3$ for $\forall\omega$, $E^{\mathrm{T}}(\omega)=2,4$ for $\forall\omega$, or $E^{\mathrm{T}}(\omega)=3,4$ for $\forall\omega$, positivity does not hold.

Appendix 2: causal RR in terms of response types

Here, we show that the RR shown in equation A10 (or, equation 8) is equivalent to the causal RR

shown in equation 1. First, by using the conventional notation of probability, the RR in equation A10 can be rewritten as

$$\frac{\sum_{j=1,2,3,4,5,6,7,8} P_{C|Dj}P_{Dj} + \sum_{j=1,2,3,4,9,10,11,12} P_{\bar{C}|Dj}P_{Dj}}{\sum_{j=1,2,5,6,9,10,13,14} P_{C|Dj}P_{Dj} + \sum_{j=1,3,5,9,11,13,15} P_{\bar{C}|Dj}P_{Dj}}$$

$$= \frac{\sum_{j=1,2,3,4,5,6,7,8} P_{Dj|C}P_{C} + \sum_{j=1,2,3,4,9,10,11,12} P_{Dj|\bar{C}}P_{\bar{C}}}{\sum_{j=1,2,5,6,9,10,13,14} P_{Dj|C}P_{C} + \sum_{j=1,3,5,9,11,13,15} P_{Dj|\bar{C}}P_{\bar{C}}}$$

$$= \frac{\sum_{j=1,2,3,4,5,6,7,8} P[D^{\mathrm{T}} = j \mid C=1]P[C=1] + \sum_{j=1,2,3,4,9,10,11,12} P[D^{\mathrm{T}} = j \mid C=0]P[C=0]}{\sum_{j=1,2,5,6,9,10,13,14} P[D^{\mathrm{T}} = j \mid C=1]P[C=1] + \sum_{j=1,3,5,9,11,13,15} P[D^{\mathrm{T}} = j \mid C=0]P[C=0]}.$$

Then, by referring to Table 2, this can be rewritten using the addition rule for probabilities as

$$\frac{P[\bigcup_{j=1,2,3,4,5,6,7,8} \{D^{\mathrm{T}} = j\} \mid C=1]P[C=1] + P[\bigcup_{j=1,2,3,4,9,10,11,12} \{D^{\mathrm{T}} = j\} \mid C=0]P[C=0]}{P[\bigcup_{j=1,2,5,6,9,10,13,14} \{D^{\mathrm{T}} = j\} \mid C=1]P[C=1] + P[\bigcup_{j=1,3,5,9,11,13,15} \{D^{\mathrm{T}} = j\} \mid C=0]P[C=0]}$$

$$= \frac{P[D_{e=1} = 1 \mid C=1]P[C=1] + P[D_{e=1} = 1 \mid C=0]P[C=0]}{P[D_{e=0} = 1 \mid C=1]P[C=1] + P[D_{e=0} = 1 \mid C=0]P[C=0]}$$

$$= \frac{P[D_{e=1} = 1]}{P[D_{e=0} = 1]},$$

which is equivalent to the causal RR in equation 1. In other words, the RR in equation A10 is an alternative notation of causal RR in terms of response types.

Appendix 3: sufficient conditions to estimate effect measures in observational studies by adjusting for confounding bias

We show that the weighted average of stratum-specific associational RRs in equation 7 is equivalent to the causal RR in equation 1 if $E \amalg D_e | C$ for $\forall e$ holds. First, to simplify the explanation, we show the RR in equation 7 by using the conventional notation of probability as

$$\frac{\left(\frac{\sum_{i=1,2;\ j=1,2,3,4,5,6,7,8} P[C=1]P[E^{\mathrm{T}} = i, D^{\mathrm{T}} = j \mid C=1]}{\sum_{i=1,2} P[E^{\mathrm{T}} = i \mid C=1]} + \frac{\sum_{i=1,3;\ j=1,2,3,4,9,10,11,12} P[C=0]P[E^{\mathrm{T}} = i, D^{\mathrm{T}} = j \mid C=0]}{\sum_{i=1,3} P[E^{\mathrm{T}} = i \mid C=0]} \right)}{\left(\frac{\sum_{i=3,4;\ j=1,2,5,6,9,10,13,14} P[C=1]P[E^{\mathrm{T}} = i, D^{\mathrm{T}} = j \mid C=1]}{\sum_{i=3,4} P[E^{\mathrm{T}} = i \mid C=1]} + \frac{\sum_{i=2,4;\ j=1,3,5,9,11,13,15} P[C=0]P[E^{\mathrm{T}} = i, D^{\mathrm{T}} = j \mid C=0]}{\sum_{i=2,4} P[E^{\mathrm{T}} = i \mid C=0]} \right)}. \quad (9)$$

Then, if $E \amalg D_e | C$ for $\forall\, e$ holds, this can be rewritten by referring to Tables 1 and 2 as

$$
\begin{aligned}
&\frac{\left(\dfrac{P[C=1] \sum\limits_{j=1,2,3,4,5,6,7,8} \sum\limits_{i=1,2} P[E^{\mathrm{T}}=i, D^{\mathrm{T}}=j \mid C=1]}{\sum\limits_{i=1,2} P[E^{\mathrm{T}}=i \mid C=1]} + \dfrac{P[C=0] \sum\limits_{j=1,2,3,4,9,10,11,12} \sum\limits_{i=1,3} P[E^{\mathrm{T}}=i, D^{\mathrm{T}}=j \mid C=0]}{\sum\limits_{i=1,3} P[E^{\mathrm{T}}=i \mid C=0]} \right)}{\left(\dfrac{P[C=1] \sum\limits_{j=1,2,5,6,9,10,13,14} \sum\limits_{i=3,4} P[E^{\mathrm{T}}=i, D^{\mathrm{T}}=j \mid C=1]}{\sum\limits_{i=3,4} P[E^{\mathrm{T}}=i \mid C=1]} + \dfrac{P[C=0] \sum\limits_{j=1,3,5,9,11,13,15} \sum\limits_{i=2,4} P[E^{\mathrm{T}}=i, D^{\mathrm{T}}=j \mid C=0]}{\sum\limits_{i=2,4} P[E^{\mathrm{T}}=i \mid C=0]} \right)} \\
&= \frac{\left(\dfrac{P[C=1] \sum\limits_{j=1,2,3,4,5,6,7,8} P[E_1=1, D^{\mathrm{T}}=j \mid C=1]}{P[E_1=1 \mid C=1]} + \dfrac{P[C=0] \sum\limits_{j=1,2,3,4,9,10,11,12} P[E_0=1, D^{\mathrm{T}}=j \mid C=0]}{P[E_0=1 \mid C=0]} \right)}{\left(\dfrac{P[C=1] \sum\limits_{j=1,2,5,6,9,10,13,14} P[E_1=0, D^{\mathrm{T}}=j \mid C=1]}{P[E_1=0 \mid C=1]} + \dfrac{P[C=0] \sum\limits_{j=1,3,5,9,11,13,15} P[E_0=0, D^{\mathrm{T}}=j \mid C=0]}{P[E_0=0 \mid C=0]} \right)} \\
&= \frac{\left(\dfrac{P[C=1] \sum\limits_{j=1,2,3,4,5,6,7,8} P[E=1, D^{\mathrm{T}}=j \mid C=1]}{P[E=1 \mid C=1]} + \dfrac{P[C=0] \sum\limits_{j=1,2,3,4,9,10,11,12} P[E=1, D^{\mathrm{T}}=j \mid C=0]}{P[E=1 \mid C=0]} \right)}{\left(\dfrac{P[C=1] \sum\limits_{j=1,2,5,6,9,10,13,14} P[E=0, D^{\mathrm{T}}=j \mid C=1]}{P[E=0 \mid C=1]} + \dfrac{P[C=0] \sum\limits_{j=1,3,5,9,11,13,15} P[E=0, D^{\mathrm{T}}=j \mid C=0]}{P[E=0 \mid C=0]} \right)} \\
&= \frac{\left(\dfrac{P[C=1]P[E=1, D_{e=1}=1 \mid C=1]}{P[E=1 \mid C=1]} + \dfrac{P[C=0]P[E=1, D_{e=1}=1 \mid C=0]}{P[E=1 \mid C=0]} \right)}{\left(\dfrac{P[C=1]P[E=0, D_{e=0}=1 \mid C=1]}{P[E=0 \mid C=1]} + \dfrac{P[C=0]P[E=0, D_{e=0}=1 \mid C=0]}{P[E=0 \mid C=0]} \right)} \\
&= \frac{\left(\dfrac{P[C=1]P[E=1 \mid C=1]P[D_{e=1}=1 \mid C=1]}{P[E=1 \mid C=1]} + \dfrac{P[C=0]P[E=1 \mid C=0]P[D_{e=1}=1 \mid C=0]}{P[E=1 \mid C=0]} \right)}{\left(\dfrac{P[C=1]P[E=0 \mid C=1]P[D_{e=0}=1 \mid C=1]}{P[E=0 \mid C=1]} + \dfrac{P[C=0]P[E=0 \mid C=0]P[D_{e=0}=1 \mid C=0]}{P[E=0 \mid C=0]} \right)} \\
&= \frac{P[C=1]P[D_{e=1}=1 \mid C=1] + P[C=0]P[D_{e=1}=1 \mid C=0]}{P[C=1]P[D_{e=0}=1 \mid C=1] + P[C=0]P[D_{e=0}=1 \mid C=0]} \\
&= \frac{P[D_{e=1}=1]}{P[D_{e=0}=1]},
\end{aligned}
$$

which is equivalent to the causal RR in equation 1. The first equation is derived from Table 1, the second equation is derived from the consistency condition, the third equation is derived from Table 2, and the fourth equation is derived from the condition $E \amalg D_e | C$ for $\forall\, e$.

Next, we show that the weighted average of stratum-specific associational RRs in equation 7 is equivalent to the causal RR in equation 1 if $E \amalg D^{\mathrm{T}} | C$ holds. Under this condition, by referring to Tables 1 and 2, equation 9 can be rewritten as

$$
\frac{\left(\frac{P[C=1]\sum_{j=1,2,3,4,5,6,7,8}\sum_{i=1,2}P[E^{\mathrm{T}}=i,D^{\mathrm{T}}=j\mid C=1]}{\sum_{i=1,2}P[E^{\mathrm{T}}=i\mid C=1]}+\frac{P[C=0]\sum_{j=1,2,3,4,9,10,11,12}\sum_{i=1,3}P[E^{\mathrm{T}}=i,D^{\mathrm{T}}=j\mid C=0]}{\sum_{i=1,3}P[E^{\mathrm{T}}=i\mid C=0]}\right)}{\left(\frac{P[C=1]\sum_{j=1,2,5,6,9,10,13,14}\sum_{i=3,4}P[E^{\mathrm{T}}=i,D^{\mathrm{T}}=j\mid C=1]}{\sum_{i=3,4}P[E^{\mathrm{T}}=i\mid C=1]}+\frac{P[C=0]\sum_{j=1,3,5,9,11,13,15}\sum_{i=2,4}P[E^{\mathrm{T}}=i,D^{\mathrm{T}}=j\mid C=0]}{\sum_{i=2,4}P[E^{\mathrm{T}}=i\mid C=0]}\right)}
$$

$$
=\frac{\left(\frac{P[C=1]\sum_{j=1,2,3,4,5,6,7,8}P[E_1=1,D^{\mathrm{T}}=j\mid C=1]}{P[E_1=1\mid C=1]}+\frac{P[C=0]\sum_{j=1,2,3,4,9,10,11,12}P[E_0=1,D^{\mathrm{T}}=j\mid C=0]}{P[E_0=1\mid C=0]}\right)}{\left(\frac{P[C=1]\sum_{j=1,2,5,6,9,10,13,14}P[E_1=0,D^{\mathrm{T}}=j\mid C=1]}{P[E_1=0\mid C=1]}+\frac{P[C=0]\sum_{j=1,3,5,9,11,13,15}P[E_0=0,D^{\mathrm{T}}=j\mid C=0]}{P[E_0=0\mid C=0]}\right)}
$$

$$
=\frac{\left(\frac{P[C=1]\sum_{j=1,2,3,4,5,6,7,8}P[E=1,D^{\mathrm{T}}=j\mid C=1]}{P[E=1\mid C=1]}+\frac{P[C=0]\sum_{j=1,2,3,4,9,10,11,12}P[E=1,D^{\mathrm{T}}=j\mid C=0]}{P[E=1\mid C=0]}\right)}{\left(\frac{P[C=1]\sum_{j=1,2,5,6,9,10,13,14}P[E=0,D^{\mathrm{T}}=j\mid C=1]}{P[E=0\mid C=1]}+\frac{P[C=0]\sum_{j=1,3,5,9,11,13,15}P[E=0,D^{\mathrm{T}}=j\mid C=0]}{P[E=0\mid C=0]}\right)}
$$

$$
=\frac{\left(\frac{P[C=1]P[E=1\mid C=1]\sum_{j=1,2,3,4,5,6,7,8}P[D^{\mathrm{T}}=j\mid C=1]}{P[E=1\mid C=1]}+\frac{P[C=0]P[E=1\mid C=0]\sum_{j=1,2,3,4,9,10,11,12}P[D^{\mathrm{T}}=j\mid C=0]}{P[E=1\mid C=0]}\right)}{\left(\frac{P[C=1]P[E=0\mid C=1]\sum_{j=1,2,5,6,9,10,13,14}P[D^{\mathrm{T}}=j\mid C=1]}{P[E=0\mid C=1]}+\frac{P[C=0]P[E=0\mid C=0]\sum_{j=1,3,5,9,11,13,15}P[D^{\mathrm{T}}=j\mid C=0]}{P[E=0\mid C=0]}\right)}
$$

$$
=\frac{P[C=1]P[D_{e=1}=1\mid C=1]+P[C=0]P[D_{e=1}=1\mid C=0]}{P[C=1]P[D_{e=0}=1\mid C=1]+P[C=0]P[D_{e=0}=1\mid C=0]}
$$

$$
=\frac{P[D_{e=1}=1]}{P[D_{e=0}=1]},
$$

which is equivalent to the causal RR in equation 1. The first equation is derived from Table 1, the second equation is derived from the consistency condition, the third equation is derived from the condition $E \amalg D^{\mathrm{T}} | C$, and the fourth equation is derived from Table 2.

Finally, we show that the weighted average of stratum-specific associational RRs in equation 7 is equivalent to the causal RR in equation 1 if $E^{\mathrm{T}} \amalg D^{\mathrm{T}} | C$ holds. By using the notation in the present study, we can analogously prove this referring to Table 2 as

$$
\frac{\left(\frac{\sum_{\substack{i=1,2\\ j=1,2,3,4,5,6,7,8}} P_C P_{Ei|C} P_{Dj|C}}{\sum_{i=1,2} P_{Ei|C}} + \frac{\sum_{\substack{i=1,3\\ j=1,2,3,4,9,10,11,12}} P_{\bar{C}} P_{Ei|\bar{C}} P_{Dj|\bar{C}}}{\sum_{i=1,3} P_{Ei|\bar{C}}}\right)}{\left(\frac{\sum_{\substack{i=3,4\\ j=1,2,5,6,9,10,13,14}} P_C P_{Ei|C} P_{Dj|C}}{\sum_{i=3,4} P_{Ei|C}} + \frac{\sum_{\substack{i=2,4\\ j=1,3,5,9,11,13,15}} P_{\bar{C}} P_{Ei|\bar{C}} P_{Dj|\bar{C}}}{\sum_{i=2,4} P_{Ei|\bar{C}}}\right)}
$$

$$
= \frac{\left(\frac{P_C \sum_{j=1,2,3,4,5,6,7,8} \left(\sum_{i=1,2} P_{Ei|C}\right) P_{Dj|C}}{\sum_{i=1,2} P_{Ei|C}} + \frac{P_{\bar{C}} \sum_{j=1,2,3,4,9,10,11,12} \left(\sum_{i=1,3} P_{Ei|\bar{C}}\right) P_{Dj|\bar{C}}}{\sum_{i=1,3} P_{Ei|\bar{C}}}\right)}{\left(\frac{P_C \sum_{j=1,2,5,6,9,10,13,14} \left(\sum_{i=3,4} P_{Ei|C}\right) P_{Dj|C}}{\sum_{i=3,4} P_{Ei|C}} + \frac{P_{\bar{C}} \sum_{j=1,3,5,9,11,13,15} \left(\sum_{i=2,4} P_{Ei|\bar{C}}\right) P_{Dj|\bar{C}}}{\sum_{i=2,4} P_{Ei|\bar{C}}}\right)}
$$

$$
= \frac{P_C \sum_{j=1,2,3,4,5,6,7,8} P_{Dj|C} + P_{\bar{C}} \sum_{j=1,2,3,4,9,10,11,12} P_{Dj|\bar{C}}}{P_C \sum_{j=1,2,5,6,9,10,13,14} P_{Dj|C} + P_{\bar{C}} \sum_{j=1,3,5,9,11,13,15} P_{Dj|\bar{C}}}
$$

$$
= \frac{P[C=1]P[D_{e=1}=1 \mid C=1] + P[C=0]P[D_{e=1}=1 \mid C=0]}{P[C=1]P[D_{e=0}=1 \mid C=1] + P[C=0]P[D_{e=0}=1 \mid C=0]}
$$

$$
= \frac{P[D_{e=1}=1]}{P[D_{e=0}=1]},
$$

which is equivalent to the causal RR in equation 1.

Appendix 4: relations between the sufficient conditions in Appendix 3

Here, we show a proof of the following inclusion relation:

$$
E^{\mathrm{T}} \coprod D^{\mathrm{T}} \mid C \Rightarrow E \coprod D^{\mathrm{T}} \mid C \Rightarrow E \coprod (D_{e=1}, D_{e=0}) \mid C \Rightarrow E \coprod D_e \mid C \text{ for } \forall e
$$

First, the inclusion relation $E^{\mathrm{T}} \coprod D^{\mathrm{T}} \mid C \Rightarrow E \coprod D^{\mathrm{T}} \mid C$ can be proved as

$$E^{\mathrm{T}} \coprod D^{\mathrm{T}} \mid C \Leftrightarrow (E_1, E_0) \coprod D^{\mathrm{T}} \mid C$$

$$\Rightarrow \begin{Bmatrix} E_1 \coprod D^{\mathrm{T}} \mid C \\ E_0 \coprod D^{\mathrm{T}} \mid C \end{Bmatrix}$$

$$\Rightarrow \begin{Bmatrix} E_1 \coprod D^{\mathrm{T}} \mid C = 1 \\ E_0 \coprod D^{\mathrm{T}} \mid C = 0 \end{Bmatrix}$$

$$\Leftrightarrow \begin{Bmatrix} E \coprod D^{\mathrm{T}} \mid C = 1 \\ E \coprod D^{\mathrm{T}} \mid C = 0 \end{Bmatrix} \Leftrightarrow E \coprod D^{\mathrm{T}} \mid C.$$

Next, note that the following equivalence relation holds:

$$E \coprod D^{\mathrm{T}} \mid C \Leftrightarrow E \coprod (D_{11}, D_{01}, D_{10}, D_{00}) \mid C$$

Then,

$$E \coprod (D_{11}, D_{01}, D_{10}, D_{00}) \mid C \Rightarrow E \coprod (D_{c1}, D_{c0}) \mid C \text{ for } \forall c$$
$$\Leftrightarrow E \coprod (D_{e=1}, D_{e=0}) \mid C \text{ for } \forall c \Rightarrow E \coprod D_e \mid C \text{ for } \forall e.$$

Thus, the following inclusion relation is proved:

$$E \coprod D^{\mathrm{T}} \mid C \Rightarrow E \coprod (D_{e=1}, D_{e=0}) \mid C \Rightarrow E \coprod D_e \mid C \text{ for } \forall e$$

This completes the proof. The inclusion relation also trivially shows that the weighted average of stratum-specific associational RRs in equation 7 is equivalent to the causal RR in equation 1 if any of $E \coprod (D_{e=1}, D_{e=0}) \mid C$, $E \coprod D^{\mathrm{T}} \mid C$, or $E^{\mathrm{T}} \coprod D^{\mathrm{T}} \mid C$ holds.

Appendix 5: assumptions of monotone treatment response and monotone treatment selection

As has been well noted [4], randomization is so highly valued because it is expected to produce exchangeability, that is, the potential outcomes of D and the observed exposure E are independent. In observational studies, however, (conditional) exchangeability is not guaranteed, and researchers are required to use their expert knowledge to enhance its plausibility. On a related issue, in the field of econometrics, assumptions of monotone treatment response (MTR) [5] and monotone treatment selection (MTS) [6] were recently introduced to compensate for the lack of randomization. Although the detail of these assumptions is beyond the scope of this paper, it is worth mentioning that the MTR assumption is equivalent to the assumption of positive monotonic effect, as discussed in this study. Meanwhile, the MTS assumption can be described by using the notation in the present study as follows:

$$P\left[D_{e=1} = 1 \mid E = 1\right] \geq P\left[D_{e=1} = 1 \mid E = 0\right]$$

and

$$P\left[D_{e=0}=1 \mid E=1\right] \geq P\left[D_{e=0}=1 \mid E=0\right].$$

Note that, although the MTR assumption is at an individual level, the MTS assumption is at a population level. Also, we should note that the MTR assumption may be relevant in randomized controlled trials as well as observational studies. By contrast, the MTS assumption is primarily relevant in observational studies, except for the situation in which adherence to treatment is not perfect in randomized controlled trials. (Notably, the MTS assumption in observational studies is related to the presence of the 3 marginally open paths between E and D^{T} in Figure 8, i.e., $E \leftarrow E^{\mathrm{T}} \leftarrow U1 \rightarrow D^{\mathrm{T}}$, $E \leftarrow E^{\mathrm{T}} \leftarrow U2 \rightarrow D^{\mathrm{T}}$, and $E \leftarrow C \leftarrow U1 \rightarrow D^{\mathrm{T}}$. Even when we condition on C, only the third path can be blocked, and E and D^{T} remain connected via the first 2 paths. Thus, structurally, the MTS assumption does not always refer to the issue of "selection." In Figure 6, there are no open paths between E and D^{T}, which demonstrates that the MTS assumption is irrelevant in randomized controlled trials if adherence to treatment is perfect. Further, when using stratified randomization of E by C as shown in Figure 7, the only open path between E and D^{T}, i.e., $E \leftarrow C \leftarrow U1 \rightarrow D^{\mathrm{T}}$, can be blocked by adjusting for C.) When the information about those who did not drop out is available in observational studies (Figure 2A), the MTS assumption can be described in terms of *EDS* response types as follows (see Figure 3):

$$P\left[D_{e=1}=1\,|\,E=1\right]=\frac{\sum\limits_{\substack{i=1,2\\ j=1,2,3,4,5,6,7,8\\ k=1,2,3,4,5,6,7,8}} P_{C|EiDjSk}P_{EiDjSk}+\sum\limits_{\substack{i=1,3\\ j=1,2,3,4,9,10,11,12\\ k=1,2,3,4,5,6,7,8}} P_{\bar{C}|EiDjSk}P_{EiDjSk}}{\sum\limits_{\substack{i=1,2\\ j=1,2,3,4,5,6,7,8\\ k=1,2,3,4,5,6,7,8}} P_{C|EiDjSk}P_{EiDjSk}+\sum\limits_{\substack{i=1,2\\ j=9,10,11,12,13,14,15,16\\ k=1,2,5,6,9,10,13,14}} P_{C|EiDjSk}P_{EiDjSk}+\sum\limits_{\substack{i=1,3\\ j=1,2,3,4,9,10,11,12\\ k=1,2,3,4,5,6,7,8}} P_{\bar{C}|EiDjSk}P_{EiDjSk}+\sum\limits_{\substack{i=1,3\\ j=5,6,7,8,13,14,15,16\\ k=1,2,5,6,9,10,13,14}} P_{\bar{C}|EiDjSk}P_{EiDjSk}}\geq$$

$$\frac{\sum\limits_{\substack{i=3,4\\ j=1,2,5,6\\ k=1,2,3,4,9,10,11,12}} P_{C|EiDjSk}P_{EiDjSk}+\sum\limits_{\substack{i=3,4\\ j=3,4,7,8\\ k=1,3,5,7,9,11,13,15}} P_{C|EiDjSk}P_{EiDjSk}+\sum\limits_{\substack{i=2,4\\ j=1,3,9,11\\ k=1,2,3,4,9,10,11,12}} P_{\bar{C}|EiDjSk}P_{EiDjSk}+\sum\limits_{\substack{i=2,4\\ j=2,4,10,12\\ k=1,3,5,7,9,11,13,15}} P_{\bar{C}|EiDjSk}P_{EiDjSk}}{\sum\limits_{\substack{i=3,4\\ j=1,2,5,6,9,10,13,14\\ k=1,2,3,4,9,10,11,12}} P_{C|EiDjSk}P_{EiDjSk}+\sum\limits_{\substack{i=3,4\\ j=3,4,7,8,11,12,15,16\\ k=1,3,5,7,9,11,13,15}} P_{C|EiDjSk}P_{EiDjSk}+\sum\limits_{\substack{i=2,4\\ j=1,3,5,7,9,11,13,15\\ k=1,2,3,4,9,10,11,12}} P_{\bar{C}|EiDjSk}P_{EiDjSk}+\sum\limits_{\substack{i=2,4\\ j=2,4,6,8,10,12,14,16\\ k=1,3,5,7,9,11,13,15}} P_{\bar{C}|EiDjSk}P_{EiDjSk}}=P\left[D_{e=1}=1\,|\,E=0\right],$$

and

$$P\left[D_{e=0}=1\,|\,E=1\right]=\frac{\sum\limits_{\substack{i=1,2\\ j=1,2,5,6\\ k=1,2,3,4,5,6,7,8}} P_{C|EiDjSk}P_{EiDjSk}+\sum\limits_{\substack{i=1,2\\ j=9,10,13,14\\ k=1,2,5,6,9,10,13,14}} P_{C|EiDjSk}P_{EiDjSk}+\sum\limits_{\substack{i=1,3\\ j=1,3,9,11\\ k=1,2,3,4,5,6,7,8}} P_{\bar{C}|EiDjSk}P_{EiDjSk}+\sum\limits_{\substack{i=1,3\\ j=5,7,13,15\\ k=1,2,5,6,9,10,13,14}} P_{\bar{C}|EiDjSk}P_{EiDjSk}}{\sum\limits_{\substack{i=1,2\\ j=1,2,3,4,5,6,7,8\\ k=1,2,3,4,5,6,7,8}} P_{C|EiDjSk}P_{EiDjSk}+\sum\limits_{\substack{i=1,2\\ j=9,10,11,12,13,14,15,16\\ k=1,2,5,6,9,10,13,14}} P_{C|EiDjSk}P_{EiDjSk}+\sum\limits_{\substack{i=1,3\\ j=1,2,3,4,9,10,11,12\\ k=1,2,3,4,5,6,7,8}} P_{\bar{C}|EiDjSk}P_{EiDjSk}+\sum\limits_{\substack{i=1,3\\ j=5,6,7,8,13,14,15,16\\ k=1,2,5,6,9,10,13,14}} P_{\bar{C}|EiDjSk}P_{EiDjSk}}\geq$$

$$\frac{\sum\limits_{\substack{i=3,4\\ j=1,2,5,6,9,10,13,14\\ k=1,2,3,4,9,10,11,12}} P_{C|EiDjSk}P_{EiDjSk}+\sum\limits_{\substack{i=2,4\\ j=1,3,5,7,9,11,13,15\\ k=1,2,3,4,9,10,11,12}} P_{\bar{C}|EiDjSk}P_{EiDjSk}}{\sum\limits_{\substack{i=3,4\\ j=1,2,5,6,9,10,13,14\\ k=1,2,3,4,9,10,11,12}} P_{C|EiDjSk}P_{EiDjSk}+\sum\limits_{\substack{i=3,4\\ j=3,4,7,8,11,12,15,16\\ k=1,3,5,7,9,11,13,15}} P_{C|EiDjSk}P_{EiDjSk}+\sum\limits_{\substack{i=2,4\\ j=1,3,5,7,9,11,13,15\\ k=1,2,3,4,9,10,11,12}} P_{\bar{C}|EiDjSk}P_{EiDjSk}+\sum\limits_{\substack{i=2,4\\ j=2,4,6,8,10,12,14,16\\ k=1,3,5,7,9,11,13,15}} P_{\bar{C}|EiDjSk}P_{EiDjSk}}=P\left[D_{e=0}=1\,|\,E=0\right].$$

The full enumeration of *EDS* response types in this study would provide new assumptions at an individual level. For example, by extending positive monotonic assumption (or, the MTR assumption) to compound potential outcomes, one may be interested in the following assumption:

$$E_1(\omega) \geq E_0(\omega) \text{ and } D_{1E_1}(\omega) \geq D_{0E_0}(\omega) \text{ and } S_{E_1 D_{1E_1}}(\omega) \geq S_{E_0 D_{0E_0}}(\omega) \text{ for } \forall \omega .$$

Under this assumption, the number of possible *EDS* response types is reduced from 1,024 to 496 (data not shown). Specifically, some may consider individuals of either of the following response types represent idiosyncratic population:

$$E_1(\omega) > E_0(\omega) \text{ and } D_{1E_1}(\omega) < D_{0E_0}(\omega) \text{ and } S_{E_1 D_{1E_1}}(\omega) = S_{E_0 D_{0E_0}}(\omega) = 1,$$

or

$$E_1(\omega) < E_0(\omega) \text{ and } D_{1E_1}(\omega) > D_{0E_0}(\omega) \text{ and } S_{E_1 D_{1E_1}}(\omega) = S_{E_0 D_{0E_0}}(\omega) = 1.$$

The former assumption holds for individuals of *E* response type 2, *D* response types 9, 11, 13, or 15, and *S* response types 1, 2, 9, or 10, whereas the latter holds for individuals of *E* response type 3, *D* response types 5, 6, 13, or 14, and *S* response types 1, 2, 9, or 10.

References

1. Hernán MA, Robins JM: **Estimating causal effects from epidemiological data**. *J Epidemiol Community Health* 2006, **60:**578-586.

2. Westreich D, Cole SR: **Invited commentary: positivity in practice**. *Am J Epidemiol* 2010, **171:**674-677.

3. Petersen ML, Porter KE, Gruber S, Wang Y, van der Laan MJ: **Diagnosing and responding to violations in the positivity assumption**. *Stat Methods Med Res* 2012, **21:**31-54.

4. Greenland S, Morgenstern H: **Confounding in health research**. *Annu Rev Public Health* 2001, **22:**189-212.

5. Manski CF: **Monotone treatment response**. *Econometrica* 1997, **65:**1311-1334.

6. Manski CF, Pepper JV: **Monotone instrumental variables: with an application to the returns to schooling**. *Econometrica* 2000, **68:**997-1010.
