# Supplementary material for: A counterfactual approach to bias and effect modification in terms of response types
Source: BMC Med Res Methodol. 2013 Jul 31;13:101. doi: 10.1186/1471-2288-13-101 (PMC3765813; doi:10.1186/1471-2288-13-101)
Supplement: Additional file 2: Tables S1 and S2 — Risk ratios in terms of response types. [file 1471-2288-13-101-S2.pdf]

Table S1 Associational risk ratios in terms of response types

Pooled associational risk ratios among those who do not drop out in observational studies (see Figures 2A and 3)

$$\mathrm{aRR}_{S=1}=\frac{\left(\dfrac{\sum\limits_{\substack{i=1,2\\ j=1,2,3,4,5,6,7,8\\ k=1,2,3,4,5,6,7,8}} P_{C|EiDjSk}P_{EiDjSk}+\sum\limits_{\substack{i=1,3\\ j=1,2,3,4,9,10,11,12\\ k=1,2,3,4,5,6,7,8}} P_{\bar{C}|EiDjSk}P_{EiDjSk}}{\sum\limits_{\substack{i=1,2\\ j=1,2,3,4,5,6,7,8\\ k=1,2,3,4,5,6,7,8}} P_{C|EiDjSk}P_{EiDjSk}+\sum\limits_{\substack{i=1,2\\ j=9,10,11,12,13,14,15,16\\ k=1,2,5,6,9,10,13,14}} P_{C|EiDjSk}P_{EiDjSk}+\sum\limits_{\substack{i=1,3\\ j=1,2,3,4,9,10,11,12\\ k=1,2,3,4,5,6,7,8}} P_{\bar{C}|EiDjSk}P_{EiDjSk}+\sum\limits_{\substack{i=1,3\\ j=5,6,7,8,13,14,15,16\\ k=1,2,5,6,9,10,13,14}} P_{\bar{C}|EiDjSk}P_{EiDjSk}}\right)}{\left(\dfrac{\sum\limits_{\substack{i=3,4\\ j=1,2,5,6,9,10,13,14\\ k=1,2,3,4,9,10,11,12}} P_{C|EiDjSk}P_{EiDjSk}+\sum\limits_{\substack{i=2,4\\ j=1,3,5,7,9,11,13,15\\ k=1,2,3,4,9,10,11,12}} P_{\bar{C}|EiDjSk}P_{EiDjSk}}{\sum\limits_{\substack{i=3,4\\ j=1,2,5,6,9,10,13,14\\ k=1,2,3,4,9,10,11,12}} P_{C|EiDjSk}P_{EiDjSk}+\sum\limits_{\substack{i=3,4\\ j=3,4,7,8,11,12,15,16\\ k=1,3,5,7,9,11,13,15}} P_{C|EiDjSk}P_{EiDjSk}+\sum\limits_{\substack{i=2,4\\ j=1,3,5,7,9,11,13,15\\ k=1,2,3,4,9,10,11,12}} P_{\bar{C}|EiDjSk}P_{EiDjSk}+\sum\limits_{\substack{i=2,4\\ j=2,4,6,8,10,12,14,16\\ k=1,3,5,7,9,11,13,15}} P_{\bar{C}|EiDjSk}P_{EiDjSk}}\right)} \quad \text{(A1)}$$

Stratum-specific associational risk ratios among those who do not drop out in observational studies (see Figures 2A and 3)

$$\mathrm{aRR}_{C=1,S=1}=\frac{\sum\limits_{\substack{i=1,2\\ j=1,2,3,4,5,6,7,8\\ k=1,2,3,4,5,6,7,8}} P_{C|EiDjSk}P_{EiDjSk}}{\sum\limits_{\substack{i=1,2\\ j=1,2,3,4,5,6,7,8\\ k=1,2,3,4,5,6,7,8}} P_{C|EiDjSk}P_{EiDjSk}+\sum\limits_{\substack{i=1,2\\ j=9,10,11,12,13,14,15,16\\ k=1,2,5,6,9,10,13,14}} P_{C|EiDjSk}P_{EiDjSk}} \Bigg/ \frac{\sum\limits_{\substack{i=3,4\\ j=1,2,5,6,9,10,13,14\\ k=1,2,3,4,9,10,11,12}} P_{C|EiDjSk}P_{EiDjSk}}{\sum\limits_{\substack{i=3,4\\ j=1,2,5,6,9,10,13,14\\ k=1,2,3,4,9,10,11,12}} P_{C|EiDjSk}P_{EiDjSk}+\sum\limits_{\substack{i=3,4\\ j=3,4,7,8,11,12,15,16\\ k=1,3,5,7,9,11,13,15}} P_{C|EiDjSk}P_{EiDjSk}} \quad \text{(A2)}$$

$$\mathrm{aRR}_{C=0,S=1}=\frac{\sum\limits_{\substack{i=1,3\\ j=1,2,3,4,9,10,11,12\\ k=1,2,3,4,5,6,7,8}} P_{\bar{C}|EiDjSk}P_{EiDjSk}}{\sum\limits_{\substack{i=1,3\\ j=1,2,3,4,9,10,11,12\\ k=1,2,3,4,5,6,7,8}} P_{\bar{C}|EiDjSk}P_{EiDjSk}+\sum\limits_{\substack{i=1,3\\ j=5,6,7,8,13,14,15,16\\ k=1,2,5,6,9,10,13,14}} P_{\bar{C}|EiDjSk}P_{EiDjSk}} \Bigg/ \frac{\sum\limits_{\substack{i=2,4\\ j=1,3,5,7,9,11,13,15\\ k=1,2,3,4,9,10,11,12}} P_{\bar{C}|EiDjSk}P_{EiDjSk}}{\sum\limits_{\substack{i=2,4\\ j=1,3,5,7,9,11,13,15\\ k=1,2,3,4,9,10,11,12}} P_{\bar{C}|EiDjSk}P_{EiDjSk}+\sum\limits_{\substack{i=2,4\\ j=2,4,6,8,10,12,14,16\\ k=1,3,5,7,9,11,13,15}} P_{\bar{C}|EiDjSk}P_{EiDjSk}} \quad \text{(A3)}$$

Pooled associational risk ratios in the total population in observational studies (see Figures 2B and 3)

$$\mathrm{aRR}=\frac{\left(\dfrac{\sum\limits_{\substack{i=1,2\\ j=1,2,3,4,5,6,7,8\\ k=1,2,3,4,5,6,7,8,9,10,11,12,13,14,15,16}} P_{C|EiDjSk}P_{EiDjSk}+\sum\limits_{\substack{i=1,3\\ j=1,2,3,4,9,10,11,12\\ k=1,2,3,4,5,6,7,8,9,10,11,12,13,14,15,16}} P_{\bar{C}|EiDjSk}P_{EiDjSk}}{\sum\limits_{\substack{i=1,2\\ j=1,2,3,4,5,6,7,8,9,10,11,12,13,14,15,16\\ k=1,2,3,4,5,6,7,8,9,10,11,12,13,14,15,16}} P_{C|EiDjSk}P_{EiDjSk}+\sum\limits_{\substack{i=1,3\\ j=1,2,3,4,5,6,7,8,9,10,11,12,13,14,15,16\\ k=1,2,3,4,5,6,7,8,9,10,11,12,13,14,15,16}} P_{\bar{C}|EiDjSk}P_{EiDjSk}}\right)}{\left(\dfrac{\sum\limits_{\substack{i=3,4\\ j=1,2,5,6,9,10,13,14\\ k=1,2,3,4,5,6,7,8,9,10,11,12,13,14,15,16}} P_{C|EiDjSk}P_{EiDjSk}+\sum\limits_{\substack{i=2,4\\ j=1,3,5,9,11,13,15\\ k=1,2,3,4,5,6,7,8,9,10,11,12,13,14,15,16}} P_{\bar{C}|EiDjSk}P_{EiDjSk}}{\sum\limits_{\substack{i=3,4\\ j=1,2,3,4,5,6,7,8,9,10,11,12,13,14,15,16\\ k=1,2,3,4,5,6,7,8,9,10,11,12,13,14,15,16}} P_{C|EiDjSk}P_{EiDjSk}+\sum\limits_{\substack{i=2,4\\ j=1,2,3,4,5,6,7,8,9,10,11,12,13,14,15,16\\ k=1,2,3,4,5,6,7,8,9,10,11,12,13,14,15,16}} P_{\bar{C}|EiDjSk}P_{EiDjSk}}\right)}$$

$$=\frac{\sum\limits_{\substack{i=1,2\\ j=1,2,3,4,5,6,7,8}} P_{C|EiDj}P_{EiDj}+\sum\limits_{\substack{i=1,3\\ j=1,2,3,4,9,10,11,12}} P_{\bar{C}|EiDj}P_{EiDj}}{\sum\limits_{i=1,2} P_{C|Ei}P_{Ei}+\sum\limits_{i=1,3} P_{\bar{C}|Ei}P_{Ei}} \Bigg/ \frac{\sum\limits_{\substack{i=3,4\\ j=1,2,5,6,9,10,13,14}} P_{C|EiDj}P_{EiDj}+\sum\limits_{\substack{i=2,4\\ j=1,3,5,9,11,13,15}} P_{\bar{C}|EiDj}P_{EiDj}}{\sum\limits_{i=3,4} P_{C|Ei}P_{Ei}+\sum\limits_{i=2,4} P_{\bar{C}|Ei}P_{Ei}} \quad \text{(A4)}$$

Stratum-specific associational risk ratios in the total population in observational studies (see Figures 2B and 3)

$$\mathrm{aRR}_{C=1}=\frac{\sum\limits_{\substack{i=1,2\\ j=1,2,3,4,5,6,7,8\\ k=1,2,3,4,5,6,7,8,9,10,11,12,13,14,15,16}} P_{C|EiDjSk}P_{EiDjSk}}{\sum\limits_{\substack{i=1,2\\ j=1,2,3,4,5,6,7,8,9,10,11,12,13,14,15,16\\ k=1,2,3,4,5,6,7,8,9,10,11,12,13,14,15,16}} P_{C|EiDjSk}P_{EiDjSk}} \Bigg/ \frac{\sum\limits_{\substack{i=3,4\\ j=1,2,5,6,9,10,13,14\\ k=1,2,3,4,5,6,7,8,9,10,11,12,13,14,15,16}} P_{C|EiDjSk}P_{EiDjSk}}{\sum\limits_{\substack{i=3,4\\ j=1,2,3,4,5,6,7,8,9,10,11,12,13,14,15,16\\ k=1,2,3,4,5,6,7,8,9,10,11,12,13,14,15,16}} P_{C|EiDjSk}P_{EiDjSk}}=\frac{\left(\dfrac{\sum\limits_{\substack{i=1,2\\ j=1,2,3,4,5,6,7,8}} P_{C|EiDj}P_{EiDj}}{\sum\limits_{i=1,2} P_{C|Ei}P_{Ei}}\right)}{\left(\dfrac{\sum\limits_{\substack{i=3,4\\ j=1,2,5,6,9,10,13,14}} P_{C|EiDj}P_{EiDj}}{\sum\limits_{i=3,4} P_{C|Ei}P_{Ei}}\right)} \quad \text{(A5)}$$

$$\mathrm{aRR}_{C=0}=\frac{\sum\limits_{\substack{i=1,3\\ j=1,2,3,4,9,10,11,12\\ k=1,2,3,4,5,6,7,8,9,10,11,12,13,14,15,16}} P_{\bar{C}|EiDjSk}P_{EiDjSk}}{\sum\limits_{\substack{i=1,3\\ j=1,2,3,4,5,6,7,8,9,10,11,12,13,14,15,16\\ k=1,2,3,4,5,6,7,8,9,10,11,12,13,14,15,16}} P_{\bar{C}|EiDjSk}P_{EiDjSk}} \Bigg/ \frac{\sum\limits_{\substack{i=2,4\\ j=1,3,5,9,11,13,15\\ k=1,2,3,4,5,6,7,8,9,10,11,12,13,14,15,16}} P_{\bar{C}|EiDjSk}P_{EiDjSk}}{\sum\limits_{\substack{i=2,4\\ j=1,2,3,4,5,6,7,8,9,10,11,12,13,14,15,16\\ k=1,2,3,4,5,6,7,8,9,10,11,12,13,14,15,16}} P_{\bar{C}|EiDjSk}P_{EiDjSk}}=\frac{\left(\dfrac{\sum\limits_{\substack{i=1,3\\ j=1,2,3,4,9,10,11,12}} P_{\bar{C}|EiDj}P_{EiDj}}{\sum\limits_{i=1,3} P_{\bar{C}|Ei}P_{Ei}}\right)}{\left(\dfrac{\sum\limits_{\substack{i=2,4\\ j=1,3,5,9,11,13,15}} P_{\bar{C}|EiDj}P_{EiDj}}{\sum\limits_{i=2,4} P_{\bar{C}|Ei}P_{Ei}}\right)} \quad \text{(A6)}$$

Abbreviation: aRR, associational risk ratio.

For the definitions of notations, see Figure 3.

Table S2 Causal risk ratios in terms of response types

Pooled causal risk ratios among those who do not drop out in randomized controlled trials (see Figures 2C and 4)

$$\mathrm{cRR}_{S=1}=\frac{\left(\dfrac{\sum\limits_{\substack{j=1,2,3,4,5,6,7,8\\k=1,2,3,4,5,6,7,8}}P_{C|DjSk}P_{DjSk}+\sum\limits_{\substack{j=1,2,3,4,9,10,11,12\\k=1,2,3,4,5,6,7,8}}P_{\bar{C}|DjSk}P_{DjSk}}{\sum\limits_{\substack{j=1,2,3,4,5,6,7,8\\k=1,2,3,4,5,6,7,8}}P_{C|DjSk}P_{DjSk}+\sum\limits_{\substack{j=9,10,11,12,13,14,15,16\\k=1,2,5,6,9,10,13,14}}P_{C|DjSk}P_{DjSk}+\sum\limits_{\substack{j=1,2,3,4,9,10,11,12\\k=1,2,3,4,5,6,7,8}}P_{\bar{C}|DjSk}P_{DjSk}+\sum\limits_{\substack{j=5,6,7,8,13,14,15,16\\k=1,2,5,6,9,10,13,14}}P_{\bar{C}|DjSk}P_{DjSk}}\right)}{\left(\dfrac{\sum\limits_{\substack{j=1,2,5,6,9,10,13,14\\k=1,2,3,4,9,10,11,12}}P_{C|DjSk}P_{DjSk}+\sum\limits_{\substack{j=1,3,5,7,9,11,13,15\\k=1,2,3,4,9,10,11,12}}P_{\bar{C}|DjSk}P_{DjSk}}{\sum\limits_{\substack{j=1,2,5,6,9,10,13,14\\k=1,2,3,4,9,10,11,12}}P_{C|DjSk}P_{DjSk}+\sum\limits_{\substack{j=3,4,7,8,11,12,15,16\\k=1,3,5,7,9,11,13,15}}P_{C|DjSk}P_{DjSk}+\sum\limits_{\substack{j=1,3,5,7,9,11,13,15\\k=1,2,3,4,9,10,11,12}}P_{\bar{C}|DjSk}P_{DjSk}+\sum\limits_{\substack{j=2,4,6,8,10,12,14,16\\k=1,3,5,7,9,11,13,15}}P_{\bar{C}|DjSk}P_{DjSk}}\right)} \quad \text{(A7)}$$

Stratum-specific causal risk ratios among those who do not drop out in randomized controlled trials (see Figures 2C and 4)

$$\mathrm{cRR}_{C=1,S=1}=\frac{\sum\limits_{\substack{j=1,2,3,4,5,6,7,8\\k=1,2,3,4,5,6,7,8}}P_{C|DjSk}P_{DjSk}}{\sum\limits_{\substack{j=1,2,3,4,5,6,7,8\\k=1,2,3,4,5,6,7,8}}P_{C|DjSk}P_{DjSk}+\sum\limits_{\substack{j=9,10,11,12,13,14,15,16\\k=1,2,5,6,9,10,13,14}}P_{C|DjSk}P_{DjSk}}\Bigg/\frac{\sum\limits_{\substack{j=1,2,5,6,9,10,13,14\\k=1,2,3,4,9,10,11,12}}P_{C|DjSk}P_{DjSk}}{\sum\limits_{\substack{j=1,2,5,6,9,10,13,14\\k=1,2,3,4,9,10,11,12}}P_{C|DjSk}P_{DjSk}+\sum\limits_{\substack{j=3,4,7,8,11,12,15,16\\k=1,3,5,7,9,11,13,15}}P_{C|DjSk}P_{DjSk}} \quad \text{(A8)}$$

$$\mathrm{cRR}_{C=0,S=1}=\frac{\sum\limits_{\substack{j=1,2,3,4,9,10,11,12\\k=1,2,3,4,5,6,7,8}}P_{\bar{C}|DjSk}P_{DjSk}}{\sum\limits_{\substack{j=1,2,3,4,9,10,11,12\\k=1,2,3,4,5,6,7,8}}P_{\bar{C}|DjSk}P_{DjSk}+\sum\limits_{\substack{j=5,6,7,8,13,14,15,16\\k=1,2,5,6,9,10,13,14}}P_{\bar{C}|DjSk}P_{DjSk}}\Bigg/\frac{\sum\limits_{\substack{j=1,3,5,7,9,11,13,15\\k=1,2,3,4,9,10,11,12}}P_{\bar{C}|DjSk}P_{DjSk}}{\sum\limits_{\substack{j=1,3,5,7,9,11,13,15\\k=1,2,3,4,9,10,11,12}}P_{\bar{C}|DjSk}P_{DjSk}+\sum\limits_{\substack{j=2,4,6,8,10,12,14,16\\k=1,3,5,7,9,11,13,15}}P_{\bar{C}|DjSk}P_{DjSk}} \quad \text{(A9)}$$

Pooled causal risk ratios in the total population in ideal randomized controlled trials (see Figures 2D and 4)

$$\mathrm{cRR}=\frac{\left(\dfrac{\sum\limits_{\substack{j=1,2,3,4,5,6,7,8\\k=1,2,3,4,5,6,7,8,9,10,11,12,13,14,15,16}}P_{C|DjSk}P_{DjSk}+\sum\limits_{\substack{j=1,2,3,4,9,10,11,12\\k=1,2,3,4,5,6,7,8,9,10,11,12,13,14,15,16}}P_{\bar{C}|DjSk}P_{DjSk}}{\sum\limits_{\substack{j=1,2,3,4,5,6,7,8,9,10,11,12,13,14,15,16\\k=1,2,3,4,5,6,7,8,9,10,11,12,13,14,15,16}}P_{DjSk}}\right)}{\left(\dfrac{\sum\limits_{\substack{j=1,2,5,6,9,10,13,14\\k=1,2,3,4,5,6,7,8,9,10,11,12,13,14,15,16}}P_{C|DjSk}P_{DjSk}+\sum\limits_{\substack{j=1,3,5,9,11,13,15\\k=1,2,3,4,5,6,7,8,9,10,11,12,13,14,15,16}}P_{\bar{C}|DjSk}P_{DjSk}}{\sum\limits_{\substack{j=1,2,3,4,5,6,7,8,9,10,11,12,13,14,15,16\\k=1,2,3,4,5,6,7,8,9,10,11,12,13,14,15,16}}P_{DjSk}}\right)}$$

$$=\frac{\sum\limits_{j=1,2,3,4,5,6,7,8}P_{C|Dj}P_{Dj}+\sum\limits_{j=1,2,3,4,9,10,11,12}P_{\bar{C}|Dj}P_{Dj}}{\sum\limits_{j=1,2,5,6,9,10,13,14}P_{C|Dj}P_{Dj}+\sum\limits_{j=1,3,5,9,11,13,15}P_{\bar{C}|Dj}P_{Dj}} \quad \text{(A10)}$$

Stratum-specific causal risk ratios in the total population in ideal randomized controlled trials (see Figures 2D and 4)

$$\mathrm{cRR}_{C=1}=\frac{\sum\limits_{\substack{j=1,2,3,4,5,6,7,8\\k=1,2,3,4,5,6,7,8,9,10,11,12,13,14,15,16}}P_{C|DjSk}P_{DjSk}}{\sum\limits_{\substack{j=1,2,3,4,5,6,7,8,9,10,11,12,13,14,15,16\\k=1,2,3,4,5,6,7,8,9,10,11,12,13,14,15,16}}P_{C|DjSk}P_{DjSk}}\Bigg/\frac{\sum\limits_{\substack{j=1,2,5,6,9,10,13,14\\k=1,2,3,4,5,6,7,8,9,10,11,12,13,14,15,16}}P_{C|DjSk}P_{DjSk}}{\sum\limits_{\substack{j=1,2,3,4,5,6,7,8,9,10,11,12,13,14,15,16\\k=1,2,3,4,5,6,7,8,9,10,11,12,13,14,15,16}}P_{C|DjSk}P_{DjSk}}=\frac{\sum\limits_{j=1,2,3,4,5,6,7,8}P_{C|Dj}P_{Dj}}{\sum\limits_{j=1,2,5,6,9,10,13,14}P_{C|Dj}P_{Dj}} \quad \text{(A11)}$$

$$\mathrm{cRR}_{C=0}=\frac{\sum\limits_{\substack{j=1,2,3,4,9,10,11,12\\k=1,2,3,4,5,6,7,8,9,10,11,12,13,14,15,16}}P_{\bar{C}|DjSk}P_{DjSk}}{\sum\limits_{\substack{j=1,2,3,4,5,6,7,8,9,10,11,12,13,14,15,16\\k=1,2,3,4,5,6,7,8,9,10,11,12,13,14,15,16}}P_{\bar{C}|DjSk}P_{DjSk}}\Bigg/\frac{\sum\limits_{\substack{j=1,3,5,9,11,13,15\\k=1,2,3,4,5,6,7,8,9,10,11,12,13,14,15,16}}P_{\bar{C}|DjSk}P_{DjSk}}{\sum\limits_{\substack{j=1,2,3,4,5,6,7,8,9,10,11,12,13,14,15,16\\k=1,2,3,4,5,6,7,8,9,10,11,12,13,14,15,16}}P_{\bar{C}|DjSk}P_{DjSk}}=\frac{\sum\limits_{j=1,2,3,4,9,10,11,12}P_{\bar{C}|Dj}P_{Dj}}{\sum\limits_{j=1,3,5,9,11,13,15}P_{\bar{C}|Dj}P_{Dj}} \quad \text{(A12)}$$

Abbreviation: cRR, causal risk ratio.

For the definitions of notations, see Figure 4.
